# Characterization of Extracellular Vesicles Secreted in Lentiviral Producing HEK293SF Cell Cultures

**DOI:** 10.3390/v13050797

**Published:** 2021-04-29

**Authors:** Aline Do Minh, Alexandra T. Star, Jacek Stupak, Kelly M. Fulton, Arsalan S. Haqqani, Jean-François Gélinas, Jianjun Li, Susan M. Twine, Amine A. Kamen

**Affiliations:** 1 Department of Bioengineering, McGill University, Montreal, QC H3A 0E9, Canada; aline.dominh@mail.mcgill.ca (A.D.M.); jean-francois.gelinas.1@umontreal.ca (J.-F.G.); 2 Human Health Therapeutics Research Centre, National Research Council Canada, Ottawa, ON K1N 5A2, Canada; Alexandra.Star@nrc-cnrc.gc.ca (A.T.S.); Jacek.Stupak@nrc-cnrc.gc.ca (J.S.); Kelly.Fulton@nrc-cnrc.gc.ca (K.M.F.); Arsalan.Haqqani@nrc-cnrc.gc.ca (A.S.H.); Jianjun.Li@nrc-cnrc.gc.ca (J.L.); Susan.Twine@nrc-cnrc.gc.ca (S.M.T.)

**Keywords:** extracellular vesicles, enveloped viruses, lentiviral vectors, exosome, proteomics, lipidomics, transcriptomics

## Abstract

Lentiviral vectors (LVs) are a powerful tool for gene and cell therapy and human embryonic kidney cells (HEK293) have been extensively used as a platform for production of these vectors. Like most cells and cellular tissues, HEK293 cells release extracellular vesicles (EVs). EVs released by cells share similar size, biophysical characteristics and even a biogenesis pathway with cell-produced enveloped viruses, making it a challenge to efficiently separate EVs from LVs. Thus, EVs co-purified with LVs during downstream processing, are considered “impurities” in the context of gene and cell therapy. A greater understanding of EVs co-purifying with LVs is needed to enable improved downstream processing. To that end, EVs from an inducible lentivirus producing cell line were studied under two conditions: non-induced and induced. EVs were identified in both conditions, with their presence confirmed by transmission electron microscopy and Western blot. EV cargos from each condition were then further characterized by a multi-omic approach. Nineteen proteins were identified by mass spectrometry as potential EV markers to differentiate EVs in LV preparations. Lipid composition of EV preparations before and after LV induction showed similar enrichment in phosphatidylserine. RNA cargos in EVs showed enrichment in transcripts involved in viral processes and binding functions. These findings provide insights on the product profile of lentiviral preparations and could support the development of improved separation strategies aimed at removing co-produced EVs.

## 1. Introduction

In the past decade, gene and cell therapies have become increasingly popular tools to treat diseases such as genetic disorders, cancer, cardiovascular disease, as well as a wide spectrum of orphan diseases [1]. Recently, the cell therapy field reported significant clinical achievements, including Chimeric Antigen Receptor T cell (CAR‑T) therapy, where the patient’s own immune cells are modified to express a surface receptor to stimulate an immune response against cancer cells [2]. While many viruses have been engineered to be used in gene and cell therapies as delivery vectors, adenovirus, adeno-associated virus (AAV) and lentiviral vectors (LV) have become dominant in the field [3].

LV have several advantages over other viral vectors [4]. Their ability to mediate long-term therapeutic transgene expression [5] makes them the ideal candidate for cell therapy. However, challenges such as achieving sufficiently high yield and suitable purity for in vivo and ex vivo clinical applications need to be addressed. This is particularly crucial for large scale productions to meet the needs of large population treatments other than orphan diseases [6]. Achieving suitable purity of LVs is challenged by the presence of extracellular vesicles (EVs) such as exosomes and small shedding microvesicles, that co-purify with LVs, because they not only share a similar size, but also many biochemical and biophysical properties [7].

EVs are cell membrane-derived vesicles that bleb from most cells and are found in most body fluids. The field of EVs has gained considerable attention in the past few years and their potential as drug delivery vehicles and biomarkers for diseases is actively investigated [8]. EVs are known to transport lipids, proteins and nucleic acids. The cargo composition of EVs depends on many features, such as cell type from which they are derived and the cell environment or medium for in vitro cultures. However, the mechanism behind cargo sorting is not well understood [9].

Databases have been created to compile data pertaining to EV characterization, such as Vesiclepedia and ExoCarta [10,11]. Furthermore, guidelines standardizing the study of EVs, known as the Minimal Information for Studies of Extracellular Vesicles (MISEV 2018) have been established. Definitive markers, however, are currently not established. EVs often contain similar elements as the cell of origin but at different levels and can therefore only be described in terms of enrichment or depletion in relation to parental cells. Additionally, EV composition depends very much on the EV subtype. For instance, Endosomal Sorting Complex Required for Transport (ESCRT) machinery proteins (ALIX, TSG101, CD63, CD81 and CD9) are highly enriched in exosomes, while MMP2 and CK18 are mostly found in shedding microvesicles. EVs also have the ability to transport ribonucleic acid (RNA). Both coding and non-coding RNA were reported in next-generation sequencing studies, revealing the presence of miRNAs in EVs’ cargo which are involved in transcription regulation, post-transcription regulation and sometimes viral defence [12]. The lipid content of EVs is also important as EVs are enclosed within a single phospholipid bilayer with the lipid composition resembling that of the cell plasma membrane. In addition, exosomes are highly enriched in glycosphingolipids, sphingomyelin, cholesterol and phosphatidylserine. EV membranes also contain lipid-raft micro-domains, which are notably involved in virus morphogenesis and budding [13].

EVs and retroviruses share a biogenesis pathway using the ESCRT machinery, they incorporate similar host cell components as well as viral components [14], and also share biophysical and biochemical properties, making their separation challenging. Typical purification methods, such as chromatography based on charge or size will be ineffective at discriminating EVs and LVs. This problem needs to be addressed since EVs are released concomitantly by the cells and, thus, will be found in lentiviral preparations. As lentiviral-mediated gene therapies are intended for human use, they are strictly regulated by health authorities and any impurities in the viral preparation have to be documented as per regulatory requirements [15]. Indeed, impurities such as host cell proteins and host cell DNA are only accepted at defined level. EVs, which contain both, would require extensive characterization in order to set appropriate product specifications.

Many studies have been conducted to characterize EVs isolated from different biological fluids, tissues and even cultured cells. However, only few studies focus on cell lines used to produce viruses for vaccination or gene and cell therapy [16,17]. Moreover, these studies centered their attention on virus-like-particles versus EVs, which influenced their choice of separation technique. Methods such as step ultracentrifugation (UC), sucrose cushion used by Venereo et al. [16], or processes involving the qEV size exclusion chromatography (SEC) column with a sample loading volume of <500 µL have very low throughputs due to the volume limitation of the techniques. Additionally, these methods are labour intensive, not controlled and, therefore, would induce high variability in the yield of isolated EVs. These processes are also not scalable to accommodate large volumes of samples when extensive analysis is required. Here, we want to emphasize the use of the human embryonic kidney (HEK293) cell line to produce viral vectors for gene and cell therapies [18]. Like most cells, HEK293 cells continuously generate EVs, which will be difficult to separate from LV concomitantly produced in these cell cultures. Therefore, gaining an understanding of the characteristics of EVs generated during LV production will provide an accurate product profile for LV-mediated gene therapies, and eventually, insights to improving the LV purification process. LV production in HEK293 cells can be achieved by different methods [19]: by transient transfection using 3 to 4 plasmids, using packaging cell lines where necessary genetic elements for the assembly and functioning of the vectors have been stably integrated, or using producer cell lines where the remaining transgene plasmid has been integrated.

In this study, we developed a scalable process to isolate EVs from cultures of an inducible HEK293 lentivirus (Clone 92) producing cell line. First, we evaluated EVs produced under no-inducing conditions to extensively characterize isolated EVs for proteomic, lipidomic, and transcriptomic content. We then compared EVs from Clone 92 cells with and without LV induction. These data shed light on markers that may be exploited to improve separation approaches used during downstream processing and subsequently increase LV purity.

## 2. Materials and Methods

### 2.1. Cell Culture of HEK293SF Cells in Suspension

As a platform for lentiviral vector (LV) production, HEK293SF cell line (abbreviated hereafter as 293SF) and a stable producer cell line developed by the National Research Council Canada (NRC), HEK293SF-LVP-CMVGFPq-92 (abbreviated hereafter as Clone 92) were used in this study [20,21]. Production of the LVR2-GFP (rHIV.VSV-g CMV GFP) vesicular stomatitis virus G (VSV-G)-pseudotyped lentiviral vector is induced in the Clone 92 cell line by the addition 1 μg/mL (*w/v*) doxycycline hyclate (Millipore Sigma, Etobicoke, ON, Canada) (from a 1 mg/mL stock in nuclease-free water) and 10 μg/mL (*w/v*) 4-isopropylbenzoic acid (cumate) (Millipore Sigma) (from a 10 mg/mL stock in ethanol absolute) to produce a third-generation self-inactivating human immunodeficiency virus (SIN HIV)-based lentiviral vector which expresses the green fluorescence protein (GFP). 293SF and Clone 92 cells were cultured in shake flasks (from 20 to 300 mL working volumes) in HyCell TransFx-H medium (GE Healthcare, Chicago, IL, USA) supplemented with 4–6 mM L‑Glutamine or GlutaMAX™ (Thermo Fisher Scientific, Waltham, MA, USA) and 0.1% Kolliphor (Millipore Sigma) without serum or antibiotics, or in HEK GM medium (Xell AG, Bielefeld, Germany) supplemented with 4–6 mM L‑Glutamine or GlutaMAX™ (Thermo Fisher Scientific). Cell growth was monitored by determining live cell density based on the principle of trypan blue dye exclusion on a Vi-Cell XR cell counter (Beckman Coulter, Brea, CA, USA). Cells were passaged twice a week by diluting to 2.0 × 10^5^ live cells per mL in fresh medium.

HEK293A cells (American Type Culture Collection, Manassas, VA, USA) were used for the gene transfer assay (GTA) [22]. They were maintained in a humidified incubator at 5% CO_2_ and 37 °C in Dulbecco′s Modified Eagle′s Medium (DMEM) (Wisent, St-Bruno, QC, Canada), supplemented with 2 mM L‑Glutamine and 5% Fetal Bovine Serum (FBS) (Corning Inc., Corning, New York, NY, USA) without antibiotics. Cells were passaged twice a week.

### 2.2. Production of Conditioned Medium Containing EVs

293SF and Clone 92 (under non-induced conditions) cell lines were cultivated and the cell density was measured every day. When the cell density reached 1 × 10^6^ cells/mL, the cells were kept in culture for 2 additional days before harvest.

### 2.3. EV Isolation

#### 2.3.1. Ultrafiltration (UF) and Size Exclusion Chromatography (SEC)

EVs in non-LV producing conditions from Clone 92 cell cultures were isolated using a combination of ultrafiltration followed by size exclusion chromatography as it was reported that this technique could yield more intact and pure particles [23,24]. The cells were first removed by centrifugation. The cell pellet was kept at −20 °C for further analysis and the supernatant was filtered through a 0.45 μm vacuum polyvinylidene fluoride (PVDF) filter (VWR, Ville Mont-Royal, QC, Canada) to remove large particles. The filtrate was then subjected to ultrafiltration and diafiltration (DF) using a Vivaflow™ 50R membrane (Sartorius) with a 100 kDa MWCO pre-flushed with MilliQ water and phosphate-buffered saline (PBS) buffer (Wisent) containing 0.005% Kolliphor. The pressure and volume were monitored throughout the process. This membrane also allowed for large scale processing with volumes up to 1.5 L and reusability. The diafiltered concentrate was then loaded onto a HiScreen™ Capto™ Core 700 SEC column (GE Healthcare) which resin exhibits both size exclusion and binding properties. The Capto Core 700 column was operated in flowthrough mode on an ÄKTA avant (GE Healthcare), providing further control and allowing large volumes to be processed. The flowthrough was collected and stored at −80 °C until further analysis. In some cases, the flowthrough was subjected to an additional concentration step using a MicroKros 10 kDa MWCO hollow fiber (Repligen, Rancho Dominguez, CA, USA) or an Amicon Ultra-4 centrifugal filter unit (Millipore Sigma).

#### 2.3.2. Ultracentrifugation

The induction of Clone 92 cell cultures with cumate and doxycycline generates LV particles which are classified as biosafety level 2 (BSL2) material. As the isolation process described earlier was specifically designed for EVs, involving open handling and use of equipment not suitable for BSL 2 material, ultracentrifugation was used in order to compare EVs in non-LV producing conditions with EVs upon induction of LV production. The supernatant of Clone 92 cell culture, with and without induction, obtained after centrifugation at 1200× *g* for 5 min, was filtered through a 0.45 µm filter and then subjected to a 100,000× *g* centrifugation for 70 min at 4 °C. The pellet was then washed with PBS and centrifuged again at 100,000× *g* for 70 min at 4 °C. The pellet was resuspended in 1 mL of PBS and stored at either 2–8 °C or −80 °C until further analysis.

### 2.4. Nomenclature

Table 1 presents the nomenclature that will be used hereafter for the purpose of clarification. As the result of Clone 92 induction with cumate and doxycycline is a mixed population of EVs and LVs, the nomenclature was chosen to highlight that fact. When designating Clone 92 EVs in general without a specific isolation method, the abbreviation ^C92^EVs will be used.

### 2.5. Quantification of Functional Viral Titer by Gene Transfer Assay (GTA)

A flow cytometry-based GTA was used to determine functional viral titer [21]. Each well of a 24-well plate was seeded with 1 × 10^5^ cells of HEK293A. After leaving the cells adhere to the plate for 5 h, the medium was removed. EV and LV samples were serially diluted in DMEM (Wisent) supplemented with 8 µg/mL of polybrene (Millipore Sigma) and incubated at 37 °C for 30 min. 200 µL of diluted sample were then added to the cells for transduction and the plates were incubated overnight at 37 °C before addition of 800 µL of fresh culture medium in each well the next day. Three days post-transduction (therefore, 48 h after medium addition), cells were harvested and run on the Accuri flow cytometer (Becton Dickinson, Franklin Lakes, NJ, USA) to quantify GFP expressing cells. Accepted values ranged between 2–20% fluorescent cells out of total cell count to avoid signal due to super infection.

### 2.6. Quantification of Total Particles by Digital Drop Polymerase Chain Reaction (ddPCR)

RNA was first extracted from LV samples using the High Pure Viral Nucleic Acid Kit (Roche, Mannheim, Germany) according to the manufacturer’s instructions. The extracted RNA was then reverse transcribed into complementary deoxyribonucleic acid (cDNA) using the iScript™ Select cDNA Synthesis Kit (Bio-Rad Laboratories, Hercules, CA, USA) according to the manufacturer’s instructions and using gene-specific primers targeted towards the woodchuck hepatitis virus posttranscriptional regulatory element (WPRE) amplifying a 589-base pair fragment. Primer sequences were: forward primer (5′-GTCCTTTCCATGGCTGCTC-3′), reverse primer (5′-CCGAAGGGACGTAGCAGA-3′) (Integrated DNA Technologies, Inc., Coralville, IA, USA). Serial dilutions of cDNA were prepared in nuclease-free water. ddPCR reactions were prepared with the QX200™ ddPCR™ EvaGreen Supermix (Bio-Rad) and the WPRE primer set. PCR mixtures (22 µL) were prepared for the QX200™ Droplet Generator (Bio-Rad), with final primer concentration of 0.8 µM. After droplet generation, the following PCR program was run: one cycle of 95 °C for 10 min; 40 cycles of 95 °C for 30 sec and 60 °C for 30 sec; followed by a final extension at 72 °C for 10 min and a 4 °C hold. PCR results were analyzed with the Droplet reader and QuantaSoft (Bio-Rad).

### 2.7. Quantification of Total Particles by Flow Virometry

A few studies used FM4-64FX and reported that the unbound fractions of the dye do not interfere with the flow cytometry measurements [25,26]. Moreover, FM4-64FX was shown to efficiently label EVs as well as the retrovirus under study [26]. Cell Trace Violet (CTV) is a similar dye to Carboxyfluoresceinsuccinimidyl ester (CFSE), which has been used in many flow cytometry studies on EVs [25,27]. CTV was reported as more efficient and it has a different fluorescence spectrum than GFP, which is helpful in avoiding crosstalk, since the samples bear GFP.

A double staining experiment was performed by labeling Clone 92 EV samples with a generic lipophilic dye, FM4-64FX (Thermo Fisher Scientific), and a protein-binding dye, Cell Trace Violet (CTV) (Thermo Fisher Scientific) according to the manufacturer’s instructions. A three-laser BD LSRFortessa™ X-20 was used for acquisition and results were analyzed by FlowJo V10.2 (FlowJo LLC, Ashland, OR, USA). 405 nm filter with 450/50 fluorescent channel, and 488 nm filter with 530/30 and 780/60 fluorescent channels were used.

For small particle detection, a Cytoflex flow cytometer (Beckman Coulter, Indianapolis, IN, USA) with a photomultiplier tube (PMT) for forward scatter detection was used. Specifications for laser wavelengths and power were as follows: 488 nm–300 mW, 525/40 fluorescent channel. Acquisition was done with CytExpert (Beckman Coulter). Samples, unless otherwise indicated, were acquired at the lowest flow rate 10 μL/min. The instrument cleaning procedure prior to acquisition was as follows: 20 min with Cleaning solution (Beckman Coulter) or 20 min with 0.1% bleach followed by 20 min with distilled water.

### 2.8. Imaging of EVs by Transmission Electron Microscopy (TEM)

EV samples were prepared for negative staining TEM imaging according to Théry et al. [28]. Imaging was done on a CM 100 Transmission Electron Microscope (Philips, Eindhoven, The Netherlands) operating at 80 kV. Briefly, 10 µL samples in 2% paraformaldehyde (PFA) were fixed on Formvar-carbon coated EM grids in 1% glutaraldehyde. Samples were then stained first in a solution of uranyl oxalate then embedded in a mixture of 4% uranyl acetate and 2% methyl cellulose for 10 min on ice. The stain was then removed by touching gently the edge of the grids on a filter paper. The grids were air dried prior to the TEM observation.

### 2.9. Immunoblot Analysis

Proteins were resolved by sodium dodecyl sulfate-polyacrylamide gel electrophoresis (SDS-PAGE), transferred to nitrocellulose membranes, blocked with 5% non-fat powdered milk in PBS-tween (PBS-T). Membranes were then probed for Western blot (WB) using antibodies against EV-enriched proteins (anti-CD9 (rabbit), anti-CD81 (mouse) and anti-TSG101 (rabbit) (Abcam, Cambridge, UK)) and against non-EV enriched proteins (anti-Calnexin (rabbit) (Cell Signaling, Danvers, MA, USA)).

### 2.10. Protein and Nucleic Acid Quantification

Protein concentration was determined using the RC/DC™ Protein Assay (Bio-Rad, Hercules, CA, USA) according to the manufacturer’s instructions.

For DNA quantification, the nucleic acids of EVs were extracted using the High Pure Viral Nucleic Acid Kit (Roche, Mannheim, Germany). Then, the DNA content was quantified with the Quant-iT™ PicoGreen™ dsDNA Assay Kit (Thermo Fisher Scientific) following the manufacturer’s instructions.

RNA extraction using the High Pure Nucleic Acid kit has been done previously [16]. This technique was however deemed not suitable for that purpose since poly(A) is used in this kit in a non-negligible concentration to precipitate the RNA. This would compromise RNA quantification since the Ribogreen kit used for total RNA quantification has a high affinity for poly(A) fractions. RNA was extracted using the Exosomal RNA Isolation Kit (Norgen, Thorold, ON, Canada). The extracted RNA was quantified with the Quant-iT™ RiboGreen™ RNA Assay Kit (Thermo Fisher Scientific) or the Qubit™ RNA assay (ThermoFisher).

### 2.11. EV Identification

Protein markers from the Minimal Information for Studies of Extracellular Vesicles 2018 (MISEV 2018) guidelines were used to confirm enrichment of EVs from their parent cells [29]. For general EV characterization, MISEV 2018 recommends showing three positive protein markers of EVs to demonstrate EV enrichment with ideally one transmembrane/lipid bound protein and one cytosolic protein. In addition to demonstrating protein enrichment, MISEV 2018 also recommends the depletion of cellular proteins using at least one negative protein marker for EVs.

### 2.12. Proteomic Analysis

#### 2.12.1. Filter-Aided Sample Preparation (FASP)

All analyses were done on three biological replicates. The samples were thawed on ice, and then boiled to ensure deactivation of the virus. Samples were subsequently aliquoted for separate proteomic and phospholipid analyses. The samples used in proteomics studies were treated with 4× lysis buffer containing 14% SDS, 400 mM Tris-HCl (pH 8.5), 400 mM dithiothreitol (DTT), and Protease Inhibitor Cocktail (Millipore Sigma). The samples were then diluted with water to reduce the lysis buffer concentration to 1X, sonicated on ice with Sonic Dismembrator (Fisher Scientific, Ottawa, ON, Canada) and subsequently boiled at 95 °C for 10 min. The samples were alkylated with 20 mM iodoacetamide and then digested using a modified filter-aided sample preparation (FASP) method [30]. Briefly, the samples were first buffer exchanged with 8 M urea using a 10 kDa MWCO filter in order to remove all detergent and alkylating reagents. A buffer exchange into 50 mM ammonium bicarbonate was then performed four times. Protein concentrations were determined by the Bradford protein assay (Bio-Rad), according to the manufacturer’s instructions. The protein suspensions were then digested with 1 µg sequencing grade modified trypsin (Promega, Madison, WI, USA) at 37 °C overnight. The resulting peptides were collected by centrifugation and acidified with formic acid (final concentration of 0.25%). The EV samples were subsequently dried down in a speed vacuum centrifuge and resuspended in 25 μL of 0.1% formic acid. Cell preparations and cell supernatants were diluted with 0.1% formic acid to yield a concentration of 0.02 μg/μL in 100 μL.

#### 2.12.2. Liquid Chromatography-Tandem Mass Spectrometry (LC MS/MS) Analysis

The acidified peptides were separated by reversed-phase liquid chromatography (RPLC) using a nanoAcquity ultra-high-performance liquid chromatography (nUPLC) (Waters, Milford, MA, USA) coupled to LTQ-Orbitrap-XL ETD mass spectrometer (Thermo Fisher Scientific) with a nano-electrospray ionization (ESI) interface operated in positive ion mode. The analysis involved injection and loading of approximately 10 µL of the peptide sample onto an inline Pepmap100 300 µm × 5 mm C8 Acclaim 5 µm 100 Å precolumn (Thermo Fisher Scientific), and Nano-Acquity Symmetry C18, 5 µm, 180 um × 2 cm Trap (Waters) followed by separation using a 100 µm I.D. × 10 cm 1.7 µm BEH130C18 nanoLC column (Waters). The mobile phase consisted of 0.1% (*v/v*) formic acid in HPLC grade water as solvent A and 0.1% (*v/v*) formic acid in acetonitrile as solvent B. The peptides were separated using a gradient ramping from 0.2% to 40% solvent B over 45 min, 40% to 95% solvent B over 4 min, and then re-equilibrating from 95% to 0.2% solvent B over 11 min at a flow rate of 500 nL/min. A 30-min clean-up gradient was run between samples to minimize carryover. Data was acquired on ions with mass/charge (*m/z*) ratio between 400 and 2000 Da in profile mode at a resolution of 60,000 in the Orbitrap followed by data-dependent analysis (DDA) MS/MS scans of the top three ions per scan using collision-induced dissociation (CID) for fragmentation and detection in the ion trap with the following settings: isolation width of 3.0, normalized collision energy of 35.0, activation Q of 0.250, and activation time of 30,000 ms.

#### 2.12.3. Mascot Database Search

The raw files generated by MS analysis were converted to mascot generic files (mgf) and mzXML files using ProteoWizard [31] (version 3.0.18250, ProteoWizard Software Foundation, Palo Alto, CA, USA). Files were submitted to Mascot search engine [32] (version 2.6.2, Matrix Science, London, United Kingdom) to search against protein sequence databases consisting of target and decoy sequences. The target sequences included the human Uniprot database [33] (release 2019) combined with HIV genome translated genome sequence and GFP sequences. The decoy database was constructed with reverse sequences from the target database. Searches were restricted to trypsin cleavage with one missed cleavage accepted. The peptide tolerance was set to ± 5 ppm with a fragment mass tolerance of ± 0.8 Da. Carbamidomethylation on cysteine residues was set as a fixed modification while oxidation of methionine residues was set as a variable modification. False discovery rate (FDR) in Mascot searching was calculated as follows:

(1) $$FDR=\frac{N_{decoy}}{N_{target}},$$

where *N_decoy_* is the number of decoy hits identified and *N_target_* is the number of target hits identified. To maximize the number of true positive peptides and minimize false positives, an *FDR* of <1% was selected, which corresponded to an average Mascot ion scores ≥40.

#### 2.12.4. Proteomics Data Processing

Proteomics data analysis involved measurement and assignment of MS intensity signal to each identified protein and was performed using MatchRx software as described previously [34]. Briefly, peak intensities of all the ions in each MS run were extracted from the mzXML files and assigned to Mascot-identified proteins using the MatchRx software using their m/z, retention times and neighbouring peak coordinates. Each MS intensity was adjusted using total median normalization as described previously [34]. For each sample, total MS intensity signal was also calculated by summing intensities of all the MS intensity signals in the run and was used to estimate fraction of MS intensity (FMSI) of each protein as follows:

(2) $$FMSI\;of\;a\;protein=\frac{sum\;of\;all\;intensities\;specific\;to\;the\;protein\;in\;the\;sample}{sum\;of\;all\;intensities\;in\;the\;sample}.$$

FMSI were used to examine the enrichment or depletion of each protein in EV fractions compared to Clone 92 cells or supernatants. Proteins showing more than two natural log difference (approximately 7-fold) were considered either enriched or depleted. Since FMSI values were calculated using MS intensities, they may not correspond to true protein abundance and hence were not used to compare levels amongst proteins.

The top 50 EV proteins were selected based on the following criteria for high confidence protein identification:

1. The protein’s Mascot score had to be ≥40 (<1% FDR) with ≥2 peptides and an FMSI fold change ≥7 compared to cells and supernatant.
2. Keratins were not included in the top 50 list as their presence can be the result of sample processing.
3. The FMSI value in SEC isolated EVs had to be >0.

Venn diagrams were generated using the BioVenn website [35]. The common proteins identified in both the ExoCarta [36] and Vesiclepedia [37] databases were used for comparison.

### 2.13. Liquid Chromatography-Mass Spectrometry (LC-MS) of Phospholipids

LC-MS was carried out using a Synapt G2-Si mass spectrometer (Waters) coupled to a Dionex3000 HPLC (Thermo Fisher Scientific) using a Waters ESI source. Separations were performed on a 50 × 1 mm internal diameter 3.5 µm Zorbax XDB-C8 column (Agilent, Santa Clara, CA, USA), Solvent A was 5:1:4 IPA:MeOH:H_2_O (0.2% Formic Acid/0.028 NH_4_OH); while solvent B was IPA (0.2% Formic Acid/0.028 NH_4_OH). The following gradient program was used: 0% solvent B over 3 min, 0–95% solvent B over 12 min, 95% solvent B over 5 min, and re-equilibration at 0% solvent B for 10 min. Phospholipids were analyzed in negative-ion mode. A rolling collision energy between 45 and 160 eV was used for automated DDA MS/MS. Data interpretation was done manually using LIPID MAPS^®^ Online Tools [38]. Data was normalized by first applying correction factors based on ionization efficiencies and response factors for each type of phospholipid, then percent compositions for each fraction were calculated.

### 2.14. Transcriptomics and Bioinformatics Analysis

The quality of the RNA was assessed with the Qubit RNA assay. The sequencing library was prepared using the SMARTer smRNA-Seq kit for Illumina (Takara Bio USA, Mountain View, CA, USA), following the manufacturer’s instructions, for miRNA samples, and the SMART-Seq v4 Ultra Low Input RNA kit (Takara Bio USA) for mRNA samples. The quality of the libraries was assessed using Qubit DNA assay (Thermo Fisher Scientific), Bioanalyzer 2100 (Agilent), and qPCR. Sequencing was performed on the NextSeq 500 system (Illumina, San Diego, CA, USA), using a 1 × 75 bp SE sequencing strategy.

The gene expression levels in each mRNA sample were evaluated by aligning reads to the human GRCh38 reference genome and following published methods [39]. The gene expression level was normalized by the number of fragments per kilobase per million mapped reads (FPKMs). Enrichment analyses were performed using the GO Enrichment Analysis tool and Metascape Express Analysis [40,41,42,43]. Protein hits were classified by protein class using the Protein Analysis Through Evolutionary Relationships (PANTHER) tool [44].

## 3. Results

### 3.1. Characterization of Clone 92 EVs in the Absence of Lentiviral Particles

Although efforts have been dedicated to segregate EVs from retrovirus particles [14,45], it is currently not possible to fully separate EVs from LVs. This is even more difficult on large scale processes. It is therefore important to understand the composition of basal EVs, meaning under non-inducing conditions, as they will constitute a subpopulation that will be found in LV preparation. Thus, the first part of the study focuses on the characterization of EVs generated by Clone 92 in the absence of lentiviral particles. As described in the materials and methods section, Clone 92 cells are cultured in suspension and serum-free medium, to avoid contamination by EVs associated with serum supplementation [28]. It is also important to note that the viability of the cell cultures was maintained and monitored above 95% at all times to avoid the presence of apoptotic bodies.

#### 3.1.1. Quantification of EVs Using GFP Signal by Flow Virometry

**Figure 1:**
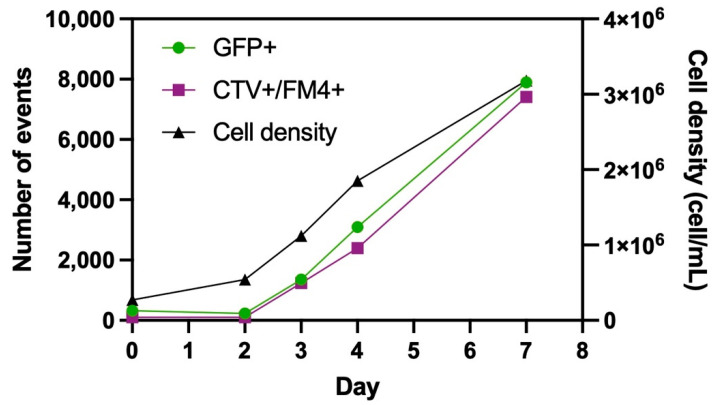
Analysis of Clone 92 supernatant by flow virometry: Quantification of GFP+ events and CTV+/FM4+ events over cell culture days as measured on the BD Fortessa flow cytometer.

Clone 92 cells express GFP constitutively, allowing the detection of particles released by the cells as Clone 92 EVs will emit a fluorescence signal. The flow virometry quantification method was first validated using a double-labeling strategy. Samples of a non-induced Clone 92 culture supernatant referred to as ^C92^EV_sup_ were taken on day 0, 2, 3, 4 and 7 and labeled with FM4-64FX and CTV. Samples were then analyzed by flow cytometry without purification. Results are presented in Figure 1.

Gating is shown in supplementary Figure S1. In Figure S1a, the gate represents GFP positive events. In Figure S1b, gating was done such as FM4-64FX positive and CTV positive events are found in quadrant Q2. In Figure S1a,b, HyCell medium serves as a negative control and shows no GFP+ signal nor FM4-64FX+/CTV+ signal before and after staining. The analysis was done on the samples mentioned above and the GFP+ events, CTV+/FM4-64FX+ events and the cell density were plotter over time on Figure 1. Figure 1 shows that GFP positive events correlated to FM4‑64FX/CTV double positive events and are increasing as the cell density increases over time. Thus, this preliminary experiment showed the feasibility of detecting ^C92^EVs using GFP fluorescence signal to enumerate the number of total particles. Subsequent flow virometry measurements were then done using only GFP signal.

Flow virometry was then used in order to estimate the number of particles bearing GFP, since GFP, which is constitutively expressed in that cell line, is being randomly incorporated into ^C92^EVs. The gating used is presented in supplementary Figure S2, PBS being used as a negative control. Samples were diluted with PBS to keep a low abort rate (ideally below 2%) and the concentrations were corrected for the dilution.

#### 3.1.2. Development of a Scalable EV Isolation Process Using Size Exclusion Chromatography (SEC)

For consistency and reproducibility, it was desirable that all analyses be performed on a single batch, thus requiring a large volume with high yield of isolated EVs to proceed with extensive characterization.

The isolation process involving UF/DF and SEC described in the materials and methods section, with or without the final concentration step, yielded EVs with an adequate volume and concentration according to the protein content and was considered as an appropriate process to produce EVs for further characterization.

This isolation process was performed 3 times and yielded 3 batched of ^C92^EV_SEC_.

Table 2 presents the mass balance of one repeat of the EV isolation process for Clone 92 culture showing recoveries at different steps in the process. Quantification was done by flow virometry in order to estimate the amount of in-process and ^C92^EV_SEC_.

Total protein quantification by RC/DC showed a reduction of 63% in the ^C92^EV_SEC_ peak as compared to the starting material. Gene transfer assay (GTA) was performed on undiluted ^C92^EV_SEC_ samples and did not show any functional titer confirming the absence of lentiviral activity.

#### 3.1.3. Preliminary Characterization Confirms EV Identity

**Figure 2:**
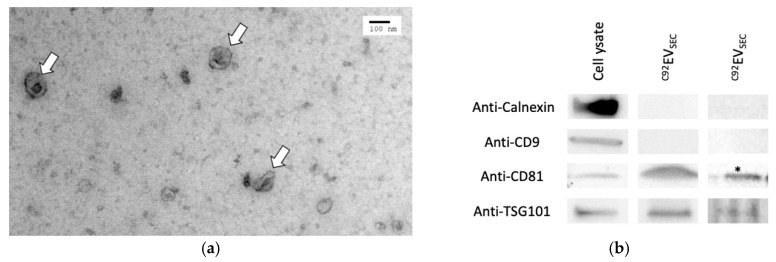
Preliminary characterization of ^C92^EV_SEC_. (**a**) Electron microscopy images of ^C92^EV_SEC_. Scale bar: 100 nm. (**b**) Enriched proteins in ^C92^EV_SEC_ identified by Western Blot. * Sample was concentrated before loading on the gel.

^C92^EV_SEC_ were imaged by transmission electron microscopy (TEM). In Figure 2a, EVs are visible as cup‑shaped indicated by white arrows. Their sizes range from about 50 to 100 nm.

CD81, TSG101 and CD9 are markers expected to be present or enriched in EVs. By WB analysis, CD81 was detected in cell lysate and ^C92^EV_SEC_ samples, with an expected enrichment in ^C92^EV_SEC_ samples (Figure 2b). TSG101 was also present in cell lysate and ^C92^EV_SEC_. The WB did not show CD9 in ^C92^EV_SEC_ samples, as the concentration of this common marker in the samples was either too low for detection or ^C92^EV_SEC_ might not be enriched in CD9. Calnexin is a protein embedded in the endoplasmic reticulum membrane and serves here as a negative marker to assess EVs purity. It was only found in cell lysate samples and not in ^C92^EV_SEC_ as expected.

#### 3.1.4. Proteomic Cargo of ^C92^EV_SEC_

Mass spectrometry (MS) was used to estimate enrichment of extracellular vesicles (EVs) by looking at the FMSI contributed by each protein to the total MS intensity in each sample. The positive identification of transmembrane proteins cluster of differentiation 81 (CD81), basigin (BSG), and the cytosolic protein, programmed cell death 6 interacting protein (PDCD6IP), confirmed the enrichment of EVs in ^C92^EV_SEC_. The FMSI of both CD81 and BSG was found to be enriched in ^C92^EV_SEC_ when compared to the Clone 92 cells, as well as the conditioned media prior to EV isolation called “supernatant” (Supplementary Figure S3). Additionally, protein PDCD6IP had a higher FMSI in the ^C92^EV_SEC_ than in the parental cells and associated supernatants (Supplementary Figure S3) suggesting enrichment in EV fractions. Endoplasmin (HSP90B1) and other heat shock proteins are good candidates as negative protein markers as they are found in the endoplasmic reticulum or mitochondria of cells and are not associated with the plasma membrane or endosomes. In this set of data, several heat shock proteins including HSP90B1, HSPD1, HSPA9, HSPE1 were depleted in ^C92^EV_SEC_ compared to the parental cells (Supplementary Figure S3). Taken together, these data indicate that the samples have been enriched for EVs.

MS was also used to detect the presence of GFP in the samples and confirm its presence in the ^C92^EV_SEC_. GFP was detected in Clone 92 cells, ^C92^EV_sup_, and ^C92^EV_SEC_ and not from the 293SF original cell line, as expected (Supplementary Figure S4). No HIV proteins were identified in any of the samples.

**Figure 3:**
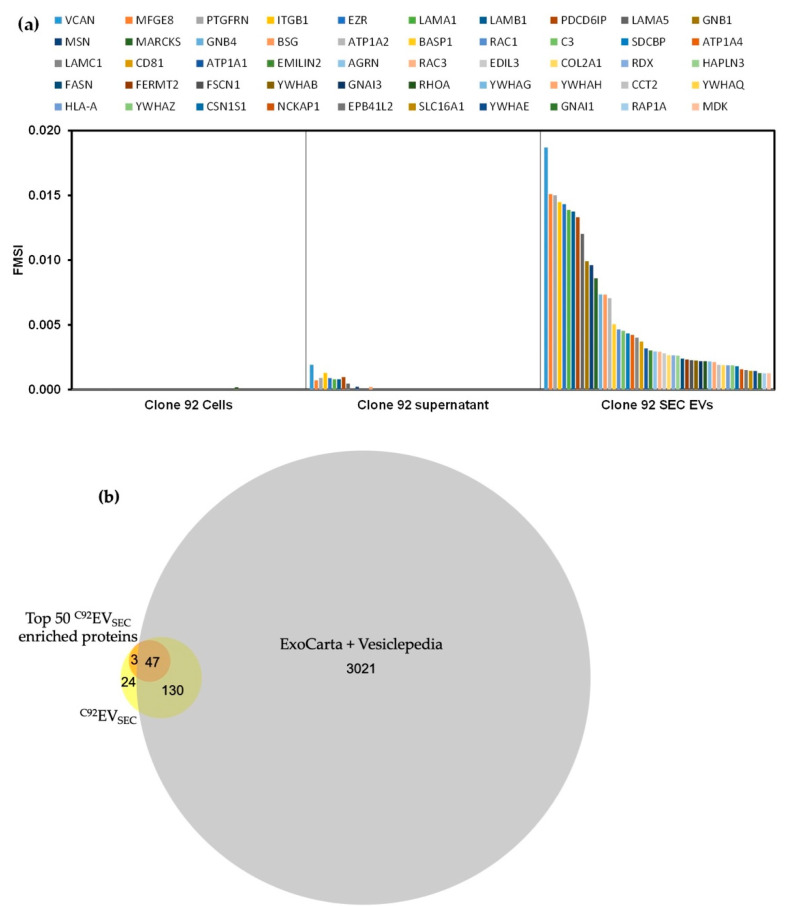
Proteomic analysis of ^C92^EV_SEC_. (**a**) Top 50 enriched proteins identified in ^C92^EV_SEC_ based on the criteria described in Materials and Methods. (**b**) Area-proportional Venn diagram for the total number of identified proteins in ^C92^EV_SEC_ and the top 50 enriched proteins in ^C92^EV_SEC_ within the combined Vesiclepedia and ExoCarta database.

The FMSI of all identified proteins in ^C92^EV_SEC_ was plotted to identify enrichment in the EV samples compared to the parental cells. Out of the 204 proteins identified, 179 showed enrichment in EVs based on their FMSI, with the top 50 of enriched proteins in ^C92^EV_SEC_ shown in Figure 3a based on their FMSI.

The total number of identified proteins in ^C92^EV_SEC_ and the top 50 enriched proteins were compared to the combined Vesiclepedia database [37] and ExoCarta database [36] in Figure 3b.

Among the total identified proteins in ^C92^EV_SEC_, 27 were not found in the combined database, and 3 of these were in the top 50 enriched proteins in ^C92^EV_SEC_: EMILIN2, MDK and ATP1A4. These proteins might be additional potential markers for ^C92^EVs.

#### 3.1.5. Lipidomic Composition of ^C92^EV_SEC_

EVs are formed by a lipid bilayer membrane. Given the size of EVs, lipids are a significant component of EVs and may play important biological roles. The field is still young; however, any data on lipids structuring EVs may give critical information related to their biogenesis.

**Figure 4:**
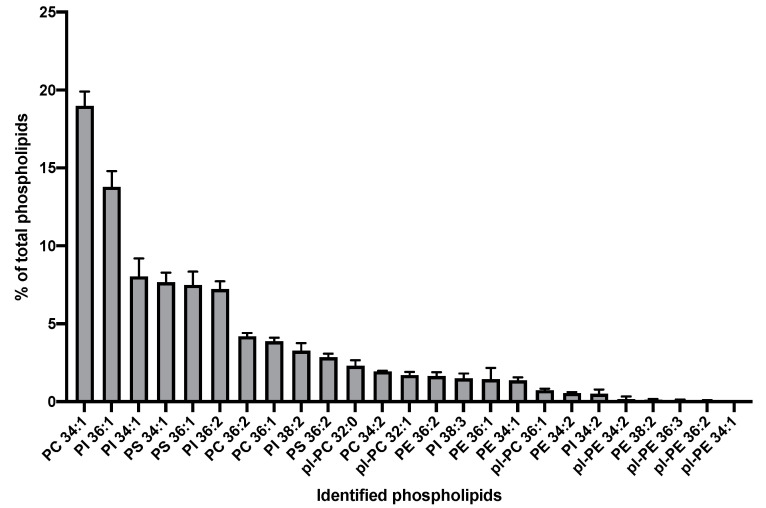
Phospholipids identified in ^C92^EV_SEC_. PC: phosphatidylcholine, PI: phosphatidylinositol, PS: phosphatidylserine, pl-PC: plasmalogen-phosphatidylcholine, PE: phosphatidylethanolamine, pl-PE: plasmalogen-phosphatidylethanolamine. Error bars indicate standard error of the mean (SEM).

The phospholipid species were quantified by liquid chromatography-mass spectrometry (LC‑MS) in three samples of ^C92^EV_SEC_ (Figure 4). LIPID MAPS consortium guidelines were followed for lipid nomenclature and the annotation of lipid species was as follows: lipid class followed by total number of carbons and degree unsaturation of respective acyl chains (e.g., PS 34:1) [46].

The most abundant phospholipids identified in ^C92^EV_SEC_ were phosphatidylcholine (PC) 34:1 and phosphatidylinositol (PI) 36:1. Hexose-ceramide (sphingolipids (SL)) were also abundantly detected at levels comparable to plasmalogen (PL), however they could not be quantified reliably.

#### 3.1.6. Nucleic Acid Content and Gene Ontology

Picogreen (DNA) and Qubit (RNA) extracted from ^C92^EV_SEC_ were performed on two different batches of ^C92^EV_SEC_ in duplicate (Table 3).

**Figure 5:**
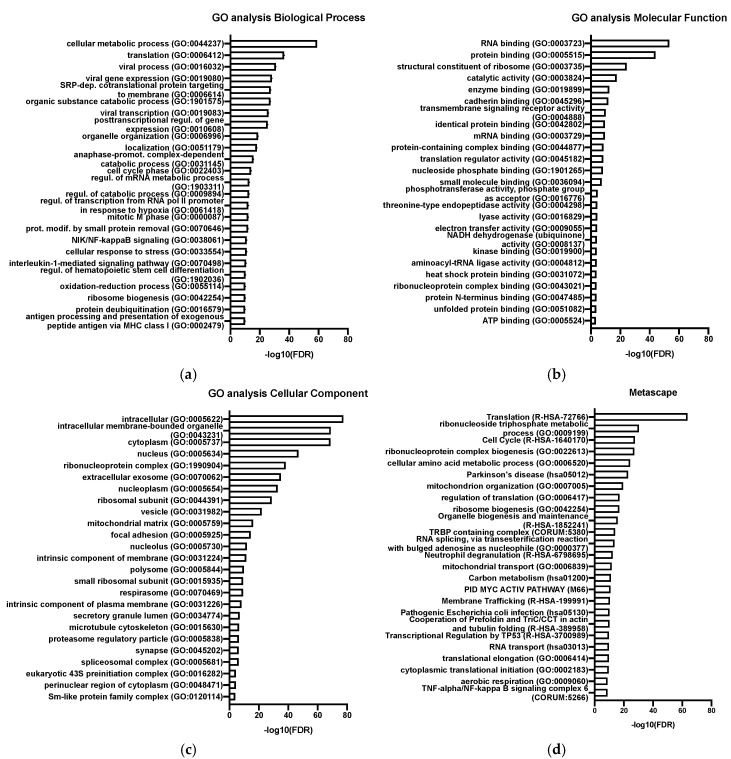
Gene ontology (GO) enrichment analysis in ^C92^EV_SEC_, top 25 ontology terms. (**a**) GO, biological process, (**b**) GO, molecular function, (**c**) GO, cellular component, (**d**) Metascape. Only terms with an FDR < 0.01 were selected.

The 3000 most expressed genes present in replicate samples of ^C92^EV_SEC_ and ranked by FPKM were analyzed for enrichment. The GO enrichment analysis tool and Metascape were both used to provide a broader search in available databases. The top 25 ontology terms are shown on Figure 5.

^C92^EV_SEC_ are enriched in genes involved in viral process, viral gene expression and viral transcription as seen in Figure 5a. The GO enrichment analysis for molecular function in Figure 5b reveals that many genes represented in EVs have a binding function such as RNA binding, protein binding and enzyme binding. Many intracellular components are abundantly found in ^C92^EV_SEC_ including intracellular membrane-bounded organelle and cytoplasm components, as well as genes associated with extracellular exosome (Figure 5c). Genes involved in DNA- and RNA-related functions are highly represented: RNA transport, viral transcription, regulation of mRNA metabolic process, transcription regulation activity, regulation of translation, etc. Other enriched genes are involved in immune system process and cellular response such as NIK/NF-kappaB signaling, anaphase-promoting complex-dependent catabolic process.

**Figure 6:**
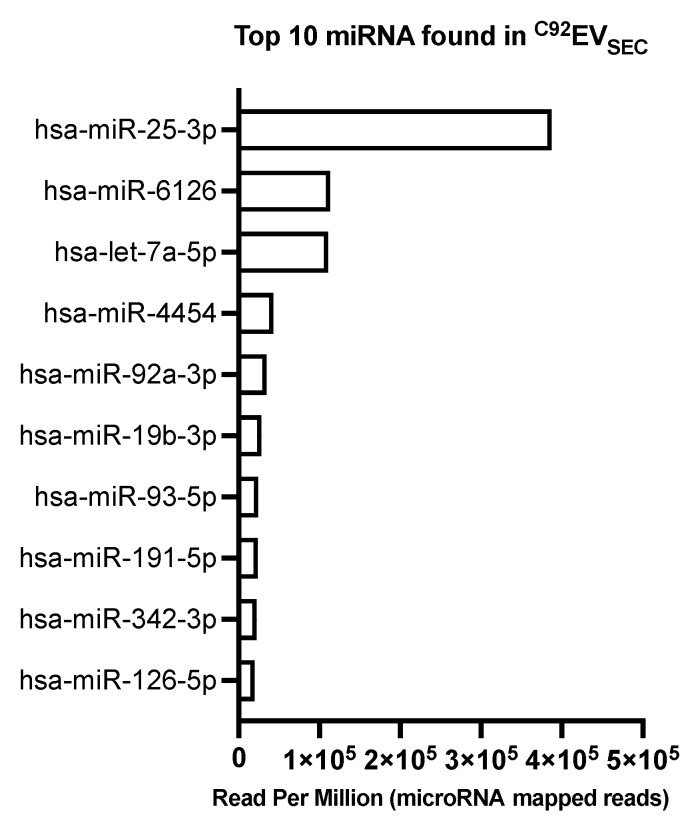
Top 10 miRNA found in ^C92^EV_SEC_.

miRNAs are highly conserved, non-coding, small single-stranded RNA molecules and have the ability to regulate gene expression. They were also characterized in ^C92^EV_SEC_. The 10 most abundant miRNAs found in ^C92^EV_SEC_ are shown in Figure 6.

The most abundant miRNA found in ^C92^EV_SEC_ was hsa-miR-25-3p, with over 3 times more read per million than the next most abundant miRNA species hsa-miR-6126 and hsa-let-7a-5p.

### 3.2. Characterization of Clone 92 EVs during Lentiviral Particles Production

As previously indicated, it is not yet feasible to effectively separate EVs from LVs in a production process. In the second part of this study, we compared EVs from Clone 92 in absence of LV induction (^C92^EV_UC_), and Clone 92 co-produced EVs following induction of LV production (^C92^EV/LV_UC_). For consistency in the sample preparations, ultracentrifugation was used as described in Section 2.3.2.

#### 3.2.1. Heterogeneity of EV and LV Populations

**Figure 7:**
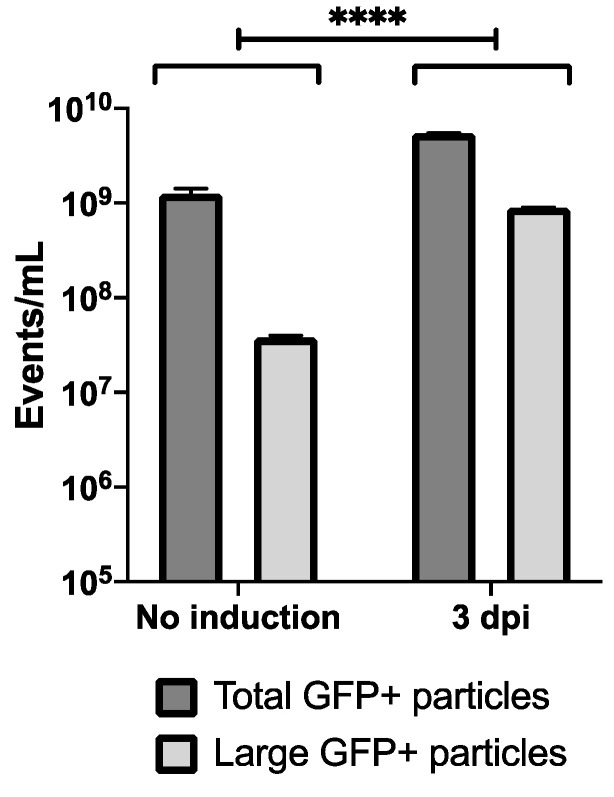
Comparison between Clone 92 supernatants with no induction and 3 days post-induction (3 dpi) by flow virometry: Quantification of flow virometry subpopulations of large particles and total GFP+ particles in each studied condition. Error bars indicate SEM. Significance is indicated by **** and is calculated via two-way ANOVA.

As flow virometry is based on GFP+ events, analysis can be performed directly on supernatant material. ^C92^EV_sup_ were therefore compared to ^C92^EV/LV_sup_ 3 days post-induction (3 dpi). Results are shown in Figure 7.

Using the same gating as in the first part of the study, total GFP+ events were higher in ^C92^EV/LV_UC_ 3 dpi. Another population was additionally observed after induction (supplementary Figure S5, still fluorescent but larger in size. A third population which was not gated in Figure S5 would include non-fluorescent even larger particles. This population was also observed in some in-process samples without induction from Table 2, suggesting large particles with no GFP but their proportion could not be estimated due to their overlap with the noise.

**Figure 8:**
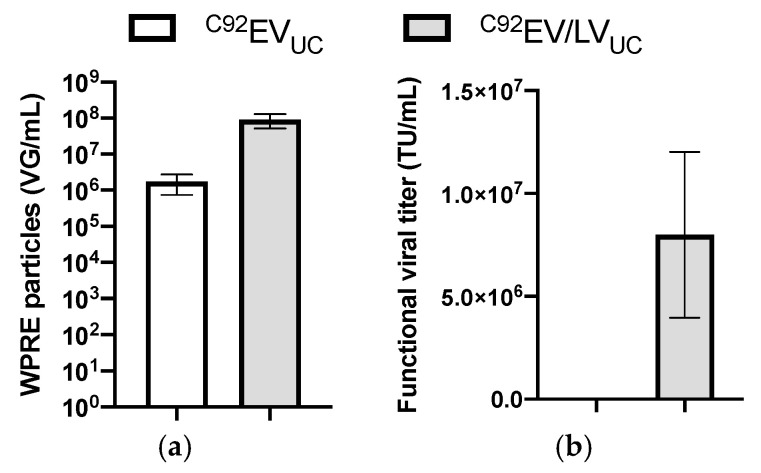
Comparison between ^C92^EV_UC_ and ^C92^EV/LV_UC_. (**a**) Quantification of WPRE particles by ddPCR. (**b**) Quantification of functional viral titer by gene transfer assay (GTA). Error bars indicate SEM.

^C92^EV_UC_ and ^C92^EV/LV_UC_ samples were analyzed by digital drop polymerase chain reaction (ddPCR) and gene transfer assay (GTA). Results are shown in Figure 8.

ddPCR allowed the quantification of particles containing the woodchuck hepatitis virus posttranscriptional regulatory element (WPRE). As seen on Figure 8a, both ^C92^EV_UC_ and ^C92^EV/LV_UC_ show a titer by ddPCR. ^C92^EV/LV_UC_’s titer is greater than ^C92^EV_UC_’s titer by two orders of magnitude.

GTA measures transgene expression (here GFP by flow cytometry) in transduced target cells to report functional viral vector particles. As in the first part of the study, ^C92^EV_UC_ samples did not show any functional titer, confirming the absence of functional LVs particles when there is no induction. ^C92^EV/LV_UC_ on the other hand confirmed the functionality of the produced LVs particles.

#### 3.2.2. Protein Cargos of EVs and LVs have Common Features

**Figure 9:**
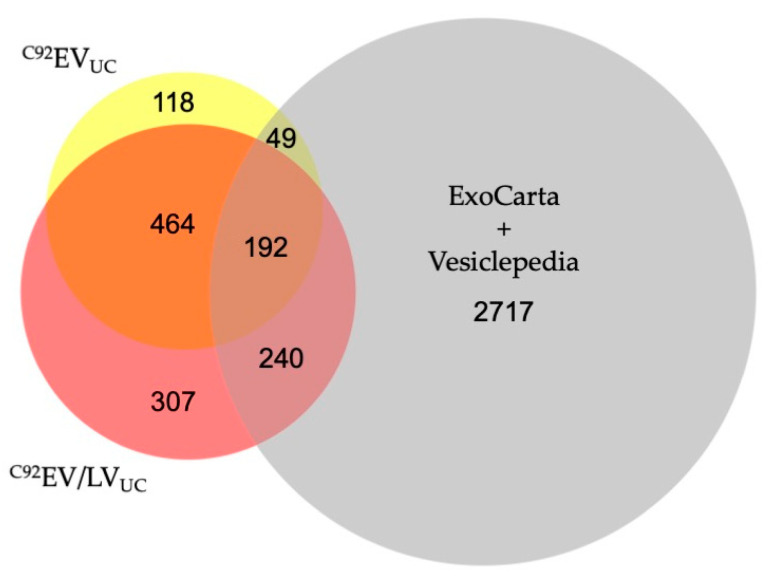
Area-proportional Venn diagram for the total number of identified proteins in ^C92^EV_UC_ and ^C92^EV/LV_UC_ within the combined Vesiclepedia and ExoCarta database.

^C92^EV_UC_ and ^C92^EV/LV_UC_ were also compared using MS. The samples contained protein markers from the MISEV 2018 guidelines: CD81 and PDCD6IP were found to be present in ^C92^EV_UC_ and ^C92^EV/LV_UC_. Additionally, prostaglandin F2 receptor inhibitor (PTGFRN), a protein from the ExoCarta database was also found in both. CD9 was not identified in the samples, consistent with ^C92^EV_SEC_ results. In addition, Calnexin and HSP90B1, common EVs “negative markers”, were not identified in any of the samples, whether under inducing or non-inducing conditions. This suggests that either EVs are indeed recovered in ^C92^EV/LV_UC_ samples or that LVs package the same proteins as EVs. A total of 822 proteins were identified in ^C92^EV_UC_ and 1203 proteins were identified in ^C92^EV/LV_UC_, with an overlap of about 48% as shown on Figure 9.

Among all the identified proteins in ^C92^EV_UC_ and ^C92^EV/LV_UC_, 167 were uniquely identified in ^C92^EV_UC_ and 548 were uniquely identified in ^C92^EV/LV_UC_ (Supplementary Table S1).

All identified proteins in ^C92^EV_UC_ and ^C92^EV/LV_UC_ were classified into 23 PANTHER protein classes (Table 4).

Metabolite interconversion enzymes and protein modifying enzymes were highly represented in all three categories (Table 4). Although also abundant in only ^C92^EV_UC_ and in both ^C92^EV_UC_ and ^C92^EV/LV_UC_, nucleic acid metabolism proteins were even more enriched in ^C92^EV/LV_UC_. Translational proteins were abundantly found in ^C92^EV/LV_UC_ only and cytoskeletal proteins were dominant in the overlap population.

Additionally, GAG-POL and VSV-G was used to identify enrichment for LV particles. Both GAG-POL and VSV-G proteins were found to be significantly more enriched in samples after LV induction in ^C92^EV/LV_UC_ and below limits of detection/identification in ^C92^EV_UC_.

GFP was identified in both ^C92^EV_UC_ and ^C92^EV/LV_UC_. Lower level of GFP was seen in samples before induction.

#### 3.2.3. Phospholipid Content in EVs and LVs

**Figure 10:**
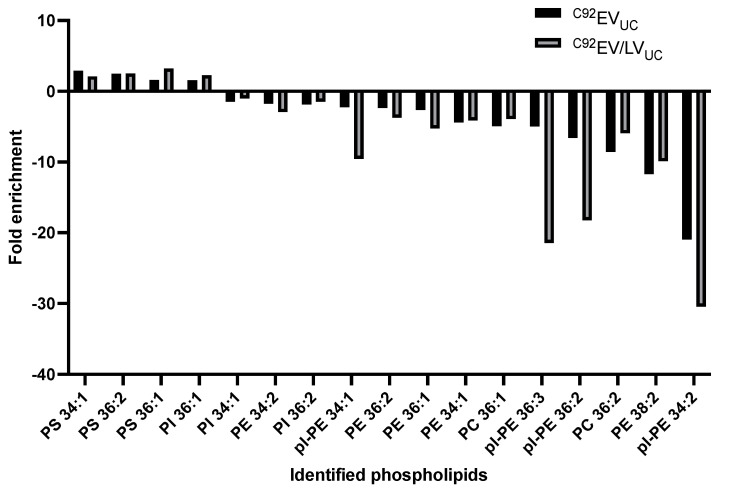
Phospholipids identified in Clone 92 EVs and LVs compared to Clone 92 parent cells. PS: phosphatidylserine, PI: phosphatidylinositol, PE: phosphatidylethanolamine, pl-PE: plasmalogen-phosphatidylethanolamine PC: phosphatidylcholine. Error bars indicate SEM.

The phospholipid species were quantified by liquid chromatography-mass spectrometry (LC‑MS) and compared between Clone 92 cells (cell pellet), ^C92^EV_UC_ and ^C92^EV/LV_UC_. The identified phospholipids in ^C92^EV_UC_ and ^C92^EV/LV_UC_ were ranked by highest positive fold change to most negative fold change compared to the parent cells (Figure 10).

Differences were not statistically significant, but some semi-quantitative observations are noted and could have biological implications. ^C92^EV_UC_ membranes and ^C92^EV/LV_UC_ membranes are enriched in the same PL compared to the cell membrane: phosphatidylserine (PS) 34:1, PS 36:2, PS 36:1 and phosphatidylinositol (PI) PI 36:1. Interestingly, ^C92^EV_UC_ and ^C92^EV/LV_UC_ are enriched and depleted in the same PL compared to their parent cell. Plasmalogen-PE (pl-PE) are 1.5 to almost 5 times more depleted in ^C92^EV/LV_UC_ than in ^C92^EV_UC_.

## 4. Discussion

EVs have gained a lot of attention in the past few years, as potential biomarkers and as drug delivery vehicles. Many studies have been carried out on EVs isolated from biofluids or even cultured cells. Yet, investigations do not report on EVs as secondary products in viral vaccines or viral vectors productions. Most cell lines, especially mammalian cell lines, are known to release EVs and cell lines used as platform for biological products are no exception. The experiments completed in this study provide a comprehensive characterization of EVs produced in HEK293SF cell lines that are widely used in viral vectors and viral vaccines production. Enveloped viruses-based products including LVs are especially targeted here for their biophysical similarities to EVs as the preparations most certainly contain both EVs and viruses. To this end a large set of experiments has been done to characterize EVs associated with an inducible HEK293SF lentivirus producing cell line (Clone 92) cultured under non-induced conditions.

The characterization of EVs is greatly impacted by the isolation method [47]. Herein a process was developed that would allow all selected analyses to be performed on one single batch of EVs for results consistency. The isolation method combining SEC and UF was selected for its scalability. Moreover, an additional advantage of developing a scalable process applicable to isolation of EVs associated with HEK293SF human cell line is the generalization of this process to multiple therapeutic products derived from the HEK293SF manufacturing platform. Indeed, EVs produced in HEK293SF cell cultures might be loaded with therapeutic cargos and used as drug delivery vehicles [48]. EVs associated with the two cell lines HEK293SF and HEK293-derived lentivirus producing cell, Clone 92 cultures were investigated. Since no significant differences were found between EVs isolated from the two cell lines and because of the intrinsic GFP labeling property of Clone 92 allowing for flow virometry measurements, these studies focused on Clone 92.

EVs reported in the literature have different cellular origins and therefore no definite markers of populations have been identified. Enriched proteins are, however, observed. In this study, although we did not discriminate between exosomes and microvesicles, only enriched proteins associated with exosomes were considered for identification. Additionally, the study focused on EVs co-produced with enveloped virus products, more specifically lentiviral vectors, consequently the size of the particles observed ranged from 80 to 100 nm, which mainly corresponds to the size of exosomes and only small microvesicles.

Other orthogonal methods are available for EV and LV quantification. However, significant discrepancies in absolute values with other techniques should be expected. For example, nanoparticle tracking analysis (NTA) is based on the Brownian motion of particles in suspension and is used to determine the size distribution of purified EVs [49] and for quantification [50]. This method lacks specificity and often leads to overestimation of the total particles measured. A method for in-process LV quantification was recently published [51] involving High-Performance Liquid Chromatography (HPLC). Although the authors optimized the method for minimizing the impact of EVs, they did acknowledge the presence of EVs in the quantification of LV particles and their proportion could not be estimated since the measure of a sample with no LV particles falls outside of the claimed linear range of the method.

The different methods used in this study highlight different features of EVs. Flow virometry results reflect the presence of GFP in ^C92^EVs. As reported, the GFP+ analysis would be a better estimate of the total particles. However, it is likely that intermediate populations that do not carry GFP or have slightly different size or granularity are excluded. Moreover, this quantification method is applicable to ^C92^EVs because of the fluorescence detection and is not applicable to EVs that do not carry GFP due to the challenges associated with signal detection which does not allow differentiating EVs from the signal background in the flow cytometer analyses. ddPCR analysis targeted WPRE as a probe. Indeed, as mentioned before, the GFP transgene and therefore the WPRE element which ensures high level transgene expression, are expressed constitutively. The quantification of WPRE therefore indicates the presence of the transgene, usually referred as “viral genome” when dealing with LVs particles. ddPCR results revealed that the “viral genome” is being incorporated in a fraction of EVs, although no viral protein or viral activity is present in EVs based on the proteomic and GTA analysis. This observation might be of interest for the design and development of therapeutic EVs for delivery of specific nucleic acid cargos. The results by flow virometry differ from the ddPCR data by at least 3 orders of magnitude in ^C92^EV_SEC_ suggesting that all EVs do not incorporate the “viral genome” sequences. The GTA and ddPCR data in LVs also reveals a difference. Indeed, the functional viral titer is lower than the VG titer as previously documented in Transfiguracion et al. [51]. This underlines the difficulty in assessing absolute quantification of EVs and LVs, but it also underlines the heterogeneous nature of EVs and LVs. In that respect, EVs and LVs are not unique populations but rather a broad distribution of populations that incorporate different cellular components. Here, the results suggest that Clone 92 LV preparations are at least composed of EVs which have incorporated the “viral genome”, EVs which do not have the “viral genome”, LVs with the viral genome but are not functional, and fully functional LV particles.

Proteomic results of ^C92^EV_SEC_ showed that GFP was indeed detected in these EVs; however, no HIV proteins were found. Although Gag-Pol is under a constitutive promoter, Rev, which is tightly regulated by the cumate switch in the design of Clone 92 [21,52], induction is required for Gag efficient expression. Thus, HIV proteins are not expected to be found in Clone 92 EVs when there is no induction by cumate and doxycycline. Results confirm here the tight regulation from the switches. Proteomic analyses of Clone 92 EVs not only confirmed EVs identity, thus validating the isolation process, but they also revealed the presence of proteins commonly found in EV databases. In fact, 47 of the top 50 proteins (Figure 3a) are known markers of EVs. These markers were also used to confirm the isolation of EVs in ^C92^EV_UC_ and ^C92^EV/LV_UC_. The absence of cellular markers CANX, HSP90B1 and HSPA5 in the two EV populations has also demonstrated EV enrichment. Nineteen proteins of interest have been identified that are common between the ^C92^EV_SEC_, ^C92^EV_UC_ and ^C92^EV/LV_UC_: FASN (fatty acid synthase), MFGE8 (lactadherin), PDCD6IP (programmed cell death 6-interacting protein), CD81 (CD81 antigen), PTGFRN (prostaglandin F2 receptor negative regulator), EZR (ezrin), ATP1A1 (sodium/potassium-transporting ATPase subunit alpha-1), YWHAQ (14-3-3 protein theta), GNB1 (guanine nucleotide binding protein G(I)/G(S)/G(T) subunit beta-1), RHOA (transforming protein RhoA), ITGB1 (integrin beta-1), MSN (moesin), YWHAG (14-3-3 protein gamma), YWHAE (14-3-3 protein epsilon), BSG (basigin), CCT2 (T-complex protein 1 subunit beta), SLC16A1 (monocarboxylate transporter 1), YWHAZ (14-3-3 protein zeta/delta), and RAC1 (ras-related C3 botulinum toxin substrate 1). These proteins have been previously identified as exosome markers in ExoCarta, which further supports their use as indicators of the presence of EVs. All nineteen of these proteins are enriched in ^C92^EV_SEC_ when compared to the Clone 92 cells and supernatant (Figure 3a). The five proteins FASN, MFGE8, PDCD6IP, CD81 and PTGFRN are found to be about equally enriched in both ^C92^EV_UC_ and ^C92^EV/LV_UC_ samples. The remaining fourteen proteins are found to be significantly enriched in the ^C92^EV/LV_UC_ when compared to ^C92^EV_UC_. This could indicate that these proteins are also present in LV particles, or there are more EVs containing these proteins being produced during LV induction as well. Future work in separating EV and LV populations will help to confirm these markers. More proteins enriched in EVs compared to the conditioned medium and parental cells were also identified (Figure 3) and could be additional potential new markers for ^C92^EVs, such as Midkine (MDK in Figure 3a), a secreted protein that regulates multiple biological processes including cell proliferation, cell adhesion, cell growth, cell survival, and cell migration [53].

Discrepancies between proteins identified in ^C92^EV_SEC_ and ^C92^EV_UC_ were observed. Only 108 proteins (~11%) overlapped between ^C92^EV_SEC_ and ^C92^EV_UC_. The lack of overlap is likely due to the difference in the EV isolation methods underlining again the importance of this step. The high percentage of protein overlap (~48%) in ^C92^EV_UC_ and ^C92^EV/LV_UC_ reinforces the observation that EVs and LVs have a lot of common features.

Interestingly, a number of proteins identified in ^C92^EV/LV_UC_ were previously reported to be associated with HIV-1 virus, including EEF1A1, a translational protein [54], NONO, a nucleic acid metabolism protein [55], GAPDH, a metabolite interconversion enzyme [56], PPIA, a protein involved in host–virus interaction [57]. NONO, GAPDH and PPIA were also found in ^C92^EV_UC_ thus indicating once again the similarities between EVs and LVs. The large number of cytoskeletal proteins in both ^C92^EV_UC_ and ^C92^EV/LV_UC_ was expected as cytoskeletal proteins have been implicated in virus transport and release [58], indicating that the budding mechanism of both LV and EV rely on cytoskeletal proteins for the translocation process.

Lipid composition of EVs has mainly been described in biological fluids but not in EVs associated with HEK292SF cell cultures [59]. ^C92^EV_UC_ and ^C92^EV/LV_UC_ share a similar lipid composition, with an enrichment in phosphatidylserine as compared to the parental cells, consistent with the findings of other studies [60]. ^C92^EV_UC_ and ^C92^EV/LV_UC_ also contained less phosphatidylcholine and phosphatidylethanolamine than their parental cells. It has been reported that the change in distribution of these lipids was involved in the budding of microvesicles [61]. Sphingolipid and cholesterol analysis in LVs/EVs samples would be a good addition to this lipidomic characterization to confirm enrichment in ceramide and cholesterol in EVs and LVs as reported in these studies on lipids involved in the budding process [60,62]. The higher depletion of plasmalogen‑PE in ^C92^EV/LV_UC_ compared to ^C92^EV_UC_ might be interesting to further study as pl-PE could play an important role in membrane dynamics and intracellular signaling [63]. Discrepancies in the lipidomic profiles observed between ^C92^EV_SEC_ and ^C92^EV_UC_ is again likely due to the difference in the EV isolation methods. Techniques for studying lipids should also be further improved to quantify more accurately lipid species, which could conduct to identifying lipid markers for Clone 92 EVs or LVs.

DNA quantification is of importance especially when it comes to biologics and viral vectors and vaccines particularly because of the stringent regulation. In the field of EVs, DNA identification is often investigated with the perspective of using them as biomarkers. Additional DNA sequencing can be expected in the future. ^C92^EV cargoes also revealed different types of RNA, including miRNA. The gene ontology analyses of ^C92^EV_SEC_ confirmed the main components and functions attributed to EVs. For instance, the abundance of genes with binding functions can explain a mechanism of cargo sorting by which RNAs will interact with specific proteins to be packaged into EVs for cell-to-cell transport. The enrichment in genes involved in viral process, viral gene expression and viral transcription can be linked to the fact that EVs and some viruses including retroviruses share the same biogenesis pathways, including the ESCRT-dependant pathway. miRNAs are highly conserved, non-coding, small single-stranded RNA molecules and have the ability to regulate gene expression. They are also involved in diseases mechanisms and have been previously identified in EVs [64]. It was therefore critical to characterize them in ^C92^EV_SEC_. Most miRNA found in ^C92^EV_SEC_ were also found in biofluids [65]. The most abundant miRNAs identified in ^C92^EV_SEC_ (Figure 6) play a role in all sort of diseases: miR-25-3p and miR-93-5p in gastric cancer [66,67], miR-19b-3p and let-7a-5p in colon cancer [68,69]. Multiple cancers showed abnormal expression of miR-92a-3p while ovarian cancer cells are suggested to release exosomes containing miR-6126 abundantly [70,71]. Some miRNAs found in ^C92^EV_SEC_ may have a positive regulating role, such as miR-93-5p in glioma or myocardial damage [72,73], miR-191-5p in lung cancer [74], or miR-342-3p in liver cancer [75]. Although it has been suggested that miRNAs are packaged into EVs as a way to dispose of excessive miRNAs, the TRBP containing complex, a member of the RNA-induced silencing complex (RISC) involved in RNA silencing [76] is also enriched in ^C92^EV_SEC_. So not only do ^C92^EVs contain miRNA but they could also provide recipient cells with the miRNA processing machinery which is needed to process those miRNAs [77]. More studies on miRNA uptake from EVs should be conducted. Until then, the effect of miRNA on recipient cells cannot be excluded given the role of miRNAs in a number of diseases.

The fact that EVs share biogenesis pathways and biophysical properties with viral products produced in cell culture platforms such as lentiviral vectors produced in HEK293SF cells and derived cell lines, supports the need to characterize host cell EVs. As discussed above, the production of viral products will induce changes to EVs. In the context of cell and gene therapy, for future in vivo gene delivery of LVs, it will be critical to further investigate EV changes and the subsequent intermediate populations upon virus production to determine accurately the product profile and specifications. The effect of co-purified EVs in LV preparations on recipient cells also needs to be evaluated. Indeed, if EVs are proven to be safe, as an associated component to enveloped viral vectors and viral vaccines, they might also have a possible adjuvanting role in the vaccine formulation.

## Figures and Tables

**Table 1:** Nomenclature for EV and LV samples in Clone 92 cell line using different isolation methods in two condition: without induction of LV production or after induction of LV production using cumate and doxycycline.

	No Induction	Induction
No isolation	^C92^EV_sup_	^C92^EV/LV_sup_
Isolation by UF/SEC	^C92^EV_SEC_	N/A ^1^
Isolation by UC	^C92^EV_UC_	^C92^EV/LV_UC_

^1^ N/A: not applicable.

**Table 2:** In-process quantification of GFP+ particles by flow virometry and total protein by RC/DC during one repeat of Clone 92 EVs isolation process.

In-Process Sample	Volume (mL)	GFP+ Particles (Part/mL)	GFP+ Particles Step Recovery (%)	Total Protein (µg/mL)
Supernatant	1478	1.37 × 10^9^	N/A ^1^	85
Supernatant after 0.45 µm filtration	1473	7.73 × 10^8^	56	73
UF/DF ^2^ product	115	4.01 × 10^9^	68	181
UF/DF ^2^ product after 0.45 µm filtration	108	2.90 × 10^9^	84	188
UF/DF ^2^ permeate	1759	2.81 × 10^8^	-	32
SEC ^3^ EV peak	101	3.15 × 10^9^	102	75
SEC ^3^ post-EV peak	8	2.99 × 10^8^	103	44
Final concentrated EVs	10	2.38 × 10^10^	74	795

^1^ N/A: not applicable, ^2^ UF/DF: ultrafiltration/diafiltration, ^3^ SEC: size exclusion chromatography.

**Table 3:** Nucleic acid quantification in ^C92^EV_SEC_.

	^C92^EV_SEC_^1^
dsDNA (µg/mL)	0.4 ± 0.1
Total RNA (µg/mL)	9.7 ± 1.7

^1^ Mean ± SD.

**Table 4:** Summary of protein classes identified in both ^C92^EV_UC_ and ^C92^EV/LV_UC_, or only in either ^C92^EV_UC_ or ^C92^EV/LV_UC_.

Category Name (Accession)	Protein Hits		
	Only in ^C92^EV_UC_	Only in ^C92^EV/LV_UC_	In both ^C92^EV_UC_ and ^C92^EV/LV_UC_
extracellular matrix protein (PC00102)	5	3	4
cytoskeletal protein (PC00085)	12	41	23
transporter (PC00227)	10	31	18
scaffold/adaptor protein (PC00226)	10	21	14
cell adhesion molecule (PC00069)	2	4	6
nucleic acid metabolism protein (PC00171)	10	57	31
intercellular signal molecule (PC00207)	1	6	3
protein-binding activity modulator (PC00095)	6	26	16
viral or transposable element protein (PC00237)	1	1	0
calcium-binding protein (PC00060)	1	5	5
gene-specific transcriptional regulator (PC00264)	11	24	14
defense/immunity protein (PC00090)	0	4	3
translational protein (PC00263)	4	55	20
metabolite interconversion enzyme (PC00262)	8	56	54
protein modifying enzyme (PC00260)	21	38	35
chromatin/chromatin-binding, or -regulatory protein (PC00077)	3	8	7
transfer/carrier protein (PC00219)	1	3	4
membrane traffic protein (PC00150)	1	13	13
chaperone (PC00072)	3	13	8
cell junction protein (PC00070)	0	2	0
structural protein (PC00211)	0	0	3
storage protein (PC00210)	0	0	1
transmembrane signal receptor (PC00197)	7	16	7

## Data Availability

Supporting data not provided in the manuscript or Supplementary Material will be made available upon request.

## Supplementary Materials

The following are available online at https://www.mdpi.com/article/10.3390/v13050797/s1, Figure S1: Analysis of Clone 92 supernatant by flow virometry, Figure S2: Flow virometry density plots showing size (violet side scatter) and green fluorescence (GFP) in the negative control PBS and an in-process Clone 92 sample (here supernatant at a dilution factor of 100), Figure S3: Enrichment of EVs shown by the increase in signal for CD81, BSG, and PDCD6IP and the depletion of cellular protein in EVs shown by a decrease in signal for HSPA5 and HSP90B1 compared to parent cells, Figure S4: Presence of GFP in cells, supernatant and EVs from Clone 92, Figure S5: Flow virometry density plots showing size (violet side scatter) and green fluorescence (GFP) in the negative control PBS and in Clone 92 supernatants, Table S1: List of all proteins identified in ^C92^EV_UC_ and ^C92^EV/LV_UC_ with a Mascot score >30 and more than 1 peptide.
