# Supplementary material for: Characterization of Extracellular Vesicles Secreted in Lentiviral Producing HEK293SF Cell Cultures
Source: Viruses. 2021 Apr 29;13(5):797. doi: 10.3390/v13050797 (PMC8145507; doi:10.3390/v13050797)
Supplement: Supplementary file 1 [file viruses-13-00797-s001.zip › viruses-1181082-supplementary.pdf]

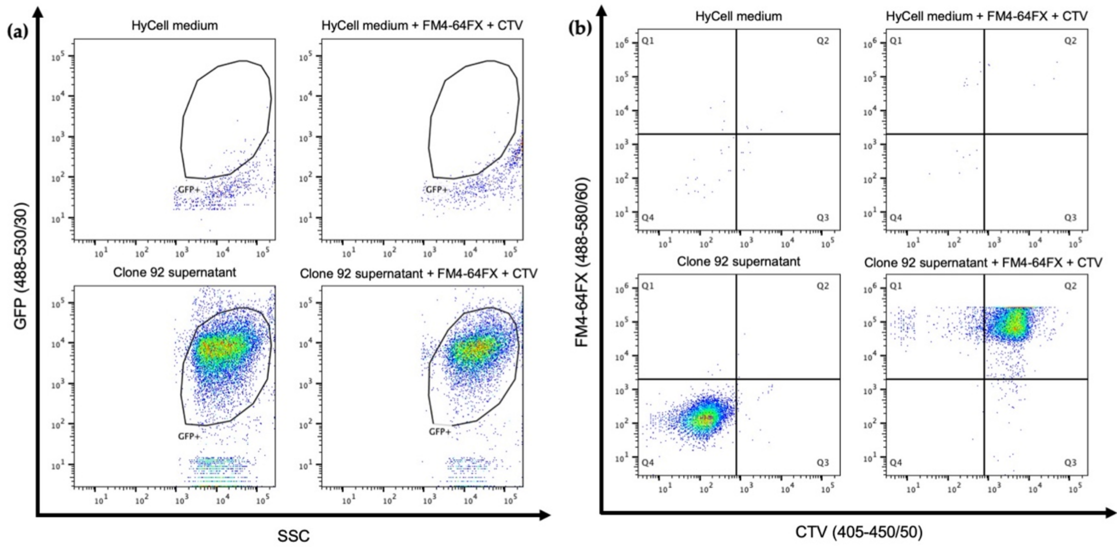

Figure S1. Analysis of Clone 92 supernatant by flow virometry. (a) GFP+ particles are depicted in the gate. The sample shown here as demonstration is $^{C92}EV_{sup}$ from day 7 of culture. (b) Double-positive events are in Q2. The sample shown here as demonstration is $^{C92}EV_{sup}$ from day 7 of culture.

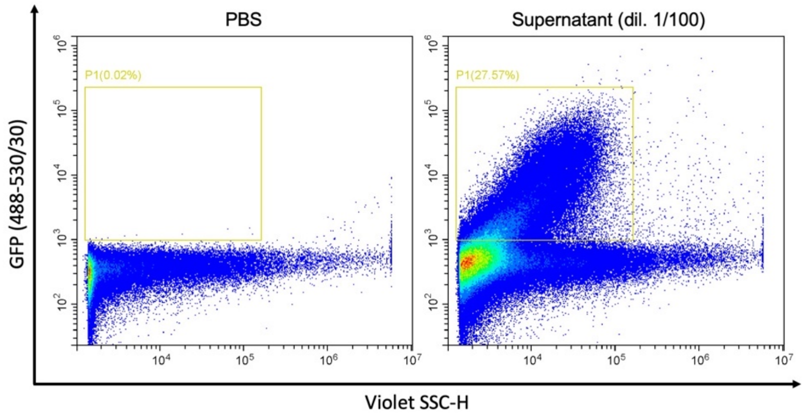

Figure S2. Flow virometry density plots showing size (violet side scatter) and green fluorescence (GFP) in the negative control PBS and an in-process Clone 92 sample (here supernatant at a dilution factor of 100). The gating P1 corresponds to GFP+ events (distribution indicated between brackets).

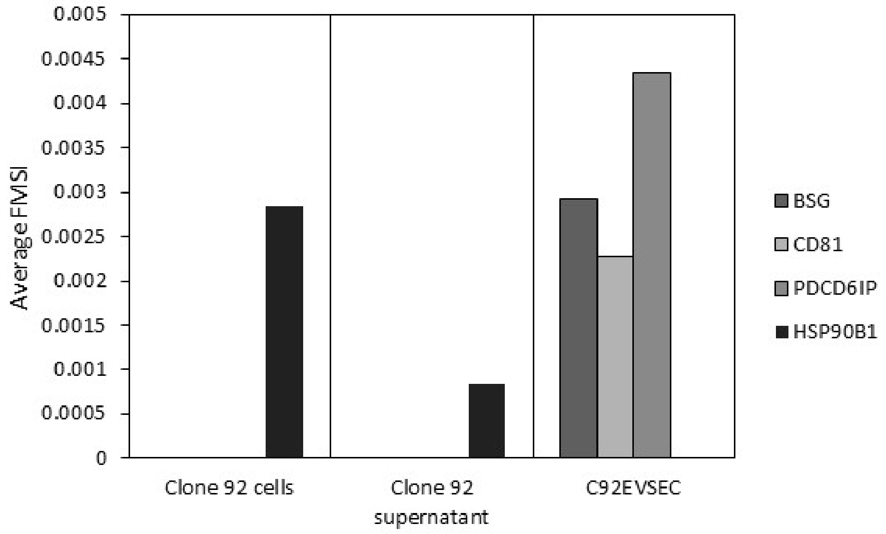

Figure S3. Enrichment of $^{C92}EV_{SEC}$ shown by the increase in signal for CD81, BSG, and PDCD6IP and the depletion of cellular protein in EVs shown by a decrease in signal for HSP90B1 compared to parent cells.

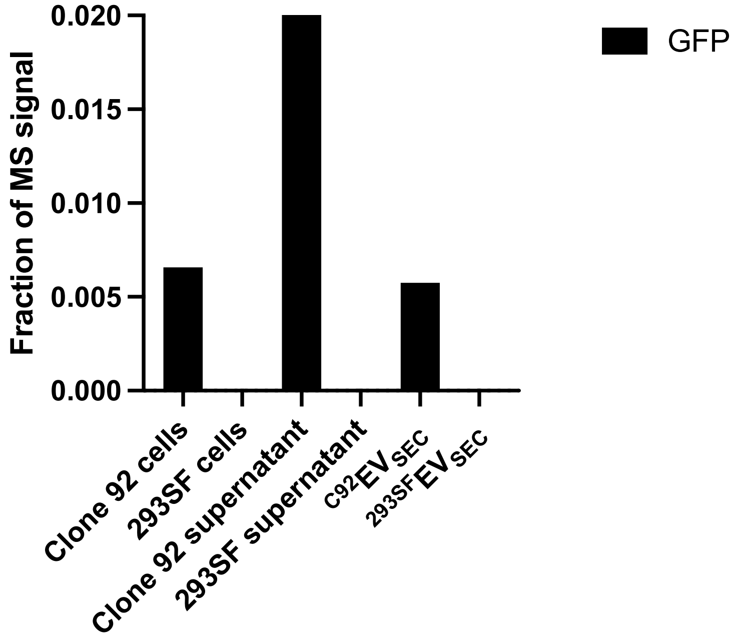

Figure S4. Presence of GFP in cells, supernatant and EVs from Clone 92.

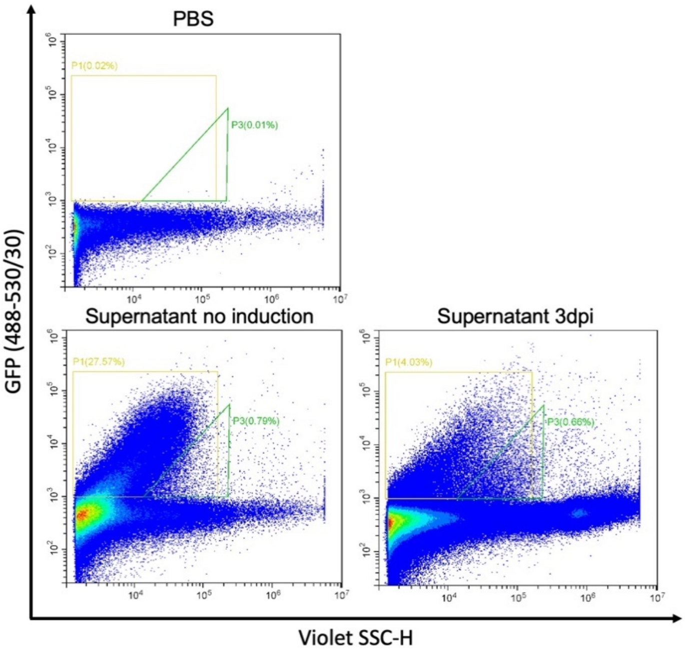

Figure S5. Flow virometry density plots showing size (violet side scatter) and green fluorescence (GFP) in the negative control PBS and in Clone 92 supernatants. The gating P1 corresponds to GFP+ events (distribution indicated between brackets) and P3 corresponds to large particles.

Table S1. List of all proteins enriched in $^{C92}EV_{UC}$ and $^{C92}EV/LV_{UC}$.

Only in $^{C92}EV_{UC}$	Only in $^{C92}EV/LV_{UC}$	In both $^{C92}EV_{UC}$ and $^{C92}EV/LV_{UC}$
AHNAK	CCT2	FASN
DGKD	PHGDH	CLTC
DMXL2	TCP1	GAG-POL
FAM120C	HNRNPU	KRT2
UTY	CCT6A	KRT1
SLC2A12	MSN	PKM
HEATR5B	CCT4	HSPA8
ATG4C	EIF4A1	KRT83
CCDC7	SFPQ	KRT10
SMC2	KPNB1	ACTG1
TRIM37	PFKP	KRT5
DNAH14	RPLP0	KRT9
MYO6	EEF1A1	TUBB
ARG1	HNRNPH1	HSPA1A
RABEP1	PFKL	KRT86
KRT40	DDX17	TUBB4B
PHLDB1	PRMT1	KRT14
USP36	ARF3	HSP90AB1
SPG11	GNB1	LGALS3BP
C1QB	HNRNPR	TUBB2B
IDI2-AS1	POTEKP	TUBA1A
COL6A2	RUVBL1	ALB
TRMT12	CCT5	MTHFD1
SRI	CTPS1	UBA1
CASC3	HNRNPM	KRT6A
NTRK3	RDX	EEF2
CD163L1	RPL18	HSP90AA1

Only in $^{C92}EV_{UC}$	Only in $^{C92}EV/LV_{UC}$	In both $^{C92}EV_{UC}$ and $^{C92}EV/LV_{UC}$
RITA1	RPS18	KRT16
TNIK	PSMD13	TUBB3
SIM1	PRDX1	ENO1
CHD1L	PSMD1	GAPDH
KCTD18	RPS6	DYNC1H1
L1TD1	H2AFV	CCT3
PTPRS	PA2G4	KRT17
CD3D	PSMD12	KRT6B
TMEM91	MAP7D3	CCT8
PCK1	DDX39B	PGK1
DZANK1	GNB2	TUBB6
FNDC8	BSG	HSPA2
ARMC3	EIF3A	HSPA1L
LOXL1	PSMD6	ATP1A1
IFNA2	PPP1CA	TUBA3E
NPPB	LDHB	ACTC1
TRIM21	NMT1	POTEE
BRCC3	HNRNPD	EZR
RBP3	RPL11	KRT85
DKK4	RPS3A	PDCD6IP
IGSF9B	PRPF19	KRT75
ZCCHC8	PPP2R1A	GFP
ANKLE2	RPL13	ATIC
CASP14	MCM4	AHCY
BRCA2	PSMB6	KRT76
MCHR1	RPS2	KRT79
MINK1	RPS8	CCT7
OR4N2	PSMC2	HSPA6
DNTTIP2	CLIC1	RACK1
LAMA5	RPL30	MYH1
LPO	CARMIL1	KRT81
THOP1	DNAH3	PPIA
PHF3	YWHAG	RPSA
RYR1	TGOLN2	RPS3
SLC26A4	SCN10A	TF
SRP54	RPL14	PTGFRN
DNAJC17	YWHAE	KRT73
ALX1	FLNA	KRT7
SNX20	ARHGEF1	CLTCL1
AOPEP	RPS15A	TTN
FBXO21	DDX5	HIST1H1D
RIC3	ARMCX5	BASP1
TMEM125	PCBP2	KRT31
BCAS3	RUVBL2	MARCKS
SLC22A10	SSRP1	KRT72
KATNB1	ARF5	EEF1G
CILP2	HNRNPA2B1	HIST1H1C
CCSER2	PSMC6	HIST1H1E
AVEN	P2RX4	KRT3
PPM1H	RPL23	KRT78
SOWAHC	BIRC6	HIST1H2BM
KLHL38	PSMA1	KRT13
NAP1L4	EPRS	HNRNPA1
SELENOW	RANBP2	KRT33A
H1F0	SAFB	KRT33B
CCNB2	RPLP2	DSP
COL1A2	IGF2BP1	ACLY
DAB2	MYH9	ATP1A2

Only in ^{C92}EV$_{UC}$	Only in ^{C92}EV/LV$_{UC}$	In both ^{C92}EV$_{UC}$ and ^{C92}EV/LV$_{UC}$
SS18L1	LDHA	ATP1A3
ZNF692	CDC42	PSMD2
ZHX3	UNC13C	NONO
OGFR	RPS20	CD81
CCDC82	RPS11	CSE1L
C10ORF90	CCDC66	CKB
PTK2B	DIS3	PSMD3
IST1	DYNC1I2	HIST3H2BB
B4GALNT4	QARS	ENO3
TPM1	MIA3	HNRNPC
MTCH1	TRAP1	HNRNPK
ASAP2	RAC1	KRT34
HMGA2	GMPS	KRT8
MAP2K7	ZNF764	APOE
PCNX1	XRCC6	HIST1H2BN
GZMM	POLG	KRT24
PPIL6	RAB1A	UBC
LMO7	APRT	RPL12
NUP188	PUM3	NPEPPS
CEMP1	CALML4	XPO1
IQGAP1	ILF2	KRT77
LXN	KAZN	KRT19
CCPG1	RPL3	PRKDC
UBTF	JAKMIP3	HNRNPL
CNN3	PCBP1	RPS5
ZNF302	NAV2	MCM2
ELK4	FAM234A	IGF2R
SERPINA3	TOPAZ1	RAN
WIZ	GLT8D2	KRT15
GRPEL1	CLIC6	ACTBL2
VIPR1	RPS17	HIST2H2AA3
POSTN	ARHGEF2	POTEI
WDR11	ADGRL2	CLU
DEPTOR	MAP10	PARP1
OR1D5	LMTK3	PLEC
KANK4	RBM44	EIF4A3
NYAP2	SON	KRT27
RGS16	CLIP2	HSPA5
DMRT2	CCDC69	LARS
SLA	TCF20	KRT35
CCDC141	PSMD7	MYH7
GIPC2	NPTN	PSMD11
ATXN7L2	DNAH6	PSMC5
FYB2	POLD1	EIF3B
C10ORF143	TNRC6B	KRT20
AASDH	ZNF853	HMGN2
TEKT2	IRF2BP1	MYH6
ST8SIA3	WDR36	MYH13
DNAI1	DDX3Y	MYH4
ATP5PD	RPL10A	MYH8
CDC37	CBR3	RBBP4
PRPF4	PDCD10	HRNR
CD82	FMR1	YWHAQ
PURG	COG5	MARCKSL1
KIF12	HES2	RHOA
RNF151	SRSF2	SPECC1L
RNF17	SRSF8	KRT71
PDCD7	FSCN1	KRT36

**Only in $^{C92}EV_{UC}$**	**Only in $^{C92}EV/LV_{UC}$**	**In both $^{C92}EV_{UC}$ and $^{C92}EV/LV_{UC}$**
KIF14	FN3KRP	KRT32
ITGB5	PSMD14	GCN1
CTTNBP2	RPL19	GNAI3
EIF4ENIF1	KDM3A	RPA1
RAG1	RPS26	LMNB2
RIBC2	ARID4B	XRCC5
KIF4A	JAM3	MUC16
RAB34	HLA-C	PCLO
SLC2A10	CMPK2	RAP1A
ACBD7	RPS10	TGM3
CBX6	PPP2CA	RPS16
CHD4	TPI1	FARSA
CSNKA2IP	SOX6	KIF22
MIPEP	ZNF644	LONP1
RPS6KC1	SF3B3	SLC1A5
TMEM249	CALB1	MYH7B
EPHA7	ASH1L	KIAA1217
TRPM7	SLC16A1	GNG12
WDR37	ZBTB39	GFAP
BACH2	ACTR1A	KRT4
MRPL39	NPBWR2	KRT80
LRRC8B	TNNI1	MUC19
ALKBH7	CFAP100	FSIP2
SLC8A2	EIF3C	LMNA
	SHTN1	CROCC
	MRPL46	NMT2
	PPA1	MDK
	RPL22	TTBK1
	RTN1	KRT12
	ZNF415	MYH3
	NPM1	LGB
	AMY1A	MFGE8
	MED25	RBMXL3
	RAI1	CFL1
	CLK1	SPEN
	BUB3	DST
	JPH3	RBBP6
	PKN2	UBA2
	NFKBIL1	PCARE
	GSTP1	SCG2
	COBLL1	NME2
	PNN	TEX10
	ARID5B	CCT6B
	CAPZA1	DARS
	SPINK5	RIMS1
	TEDC2	CABIN1
	RPL4	ALKBH8
	FGFR2	DPP6
	FGFR4	ANK3
	PRX	STRN3
	TDO2	ZNF469
	ZKSCAN5	KRT37
	HNRNPA3	CHD7
	LEO1	COL4A1
	RHOU	ANXA1
	CCER1	HMGN1
	GART	ANXA2
	JPH1	MYO9A

Only in $^{C92}EV_{UC}$	Only in $^{C92}EV/LV_{UC}$	In both $^{C92}EV_{UC}$ and $^{C92}EV/LV_{UC}$
	IGF2BP3	MYO15A
	PTPRZ1	TRIM28
	MPHOSPH9	KCNMA1
	ITGB1	SOX11
	KANK3	CACNA1E
	INPP4A	NASP
	LNP1	COL23A1
	POMC	AP4E1
	YARS	CCDC88B
	RPL36	COPB1
	RTFDC1	NOP53
	STYK1	ZZEF1
	RP1L1	ALDOA
	YWHAZ	TRIP12
	PCF11	TERT
	FAM129C	CENPE
	HIVEP1	LRRK2
	SPATA31D1	FLG
	DENND3	DCD
	RABEP2	MAP1B
	ARCN1	RPL29
	ASL	LRRC47
	MSH3	GNAI1
	PRDX6	RNF213
	ATN1	RAB11FIP1
	STOM	EIF3F
	ABT1	HYDIN
	IQUB	CSN1S1
	RAD50	DSG1
	NAA38	JMJD1C
	COL9A3	ATAD5
	SCD5	KRT222
	USP31	CCDC73
	CCDC83	SYNPO2
	RAPH1	TOP2A
	DHX15	GNG5
	ALOX12	ARHGAP23
	FGF4	GNAS
	NEXMIF	KMT2D
	CEP104	CIT
	RBBP8	SFSWAP
	KDM4D	STK33
	PLIN2	KRT23
	EXOC8	SETD1A
	TRIM77	COBL
	PTGES3	VCL
	SCAF4	CNOT11
	TJP2	DOK1
	DMAC2	LONRF3
	GPALPP1	LACTB
	SLF1	LZTS3
	ERP44	KRT18
	PYGM	MECOM
	VPS11	ITPR3
	SLC6A3	KIAA1107
	APOH	PTPRT
	DTX3L	HLA-A
	PSRC1	KCTD10

Only in $^{C92}EV_{UC}$	Only in $^{C92}EV/LV_{UC}$	In both $^{C92}EV_{UC}$ and $^{C92}EV/LV_{UC}$
	EID2	TNFAIP1
	PLXNB1	OXNAD1
	NARS	DNAJA4
	HPCAL4	CENPJ
	MYH10	RRBP1
	DIAPH2	TALDO1
	FAT3	GOLIM4
	PES1	COL25A1
	ADGRL1	TPR
	SCAMP3	TUSC1
	ACAT2	PPP1R12C
	NOP58	RPS4X
	SHROOM1	PDIA4
	ANLN	WDR87
	UNC13D	DNAH7
	EIF2B4	SPAST
	RPL7A	RPS13
	RAB15	EIF3L
	VCP	POLQ
	S100A8	EEF2K
	RPL24	MRPS5
	GNA12	PTPRB
	G3BP1	MTERF3
	SERBP1	VAMP3
	RPS24	PLXNB3
	MYL6B	TDRD15
	MCM5	PLK1
	ABCG1	ENOX1
	PDZD8	OPTN
	RPS12	CGNL1
	PVR	RNF6
	ATP6V1E1	DNAH11
	CALM2	KIF5C
	C6ORF222	CCDC63
	RPS25	EMILIN3
	NEK5	NUP50
	SNRPB2	RRM1
	PABPC4	NCAPD3
	ATP5PB	LMF1
	SLC25A21	QRICH2
	RAB7A	PKLR
	KCNV2	TMEM132A
	LAPTM4B	VIM
	MEX3B	RPL23A
	RFESD	ATP5PF
	HNRNPDL	ENTHD1
	MPHOSPH8	RPS14
	TMEM41B	PCDHB3
	IPO5	KCTD16
	AIMP2	SRRT
	SH3BP4	ZNHIT2
	LDLR	AGAP9
	FBXO10	EIF2B2
	PTH	EIF3H
	RRAS2	M6PR
	CD58	CARD14
	FHL1	AKR7A2
	FAM184A	MUCL3

Only in $^{C92}EV_{UC}$	Only in $^{C92}EV/LV_{UC}$	In both $^{C92}EV_{UC}$ and $^{C92}EV/LV_{UC}$
	PSMC4	ITGA6
	MYLK4	DCDC2B
	DIS3L2	TMTC1
	IRAK2	TSPAN7
	CCDC142	RNF168
	IMPDH2	CWF19L2
	IRF8	ZNF397
	CLASP1	ALAD
	DDAH1	SRCIN1
	OR2T8	ZMAT3
	MRM3	TECTA
	FAM53A	ADAM15
	DKC1	PROCA1
	CCDC12	F13A1
	SYNM	C1ORF94
	RIC8B	PRC1
	TRIM7	DDX1
	DOCK2	CSN1S2
	GIMAP6	SHROOM3
	C3ORF49	EYS
	HACL1	NFX1
	EP400	FLG2
	F11R	SLC22A7
	NUMA1	RAB21
	MALT1	SMARCA5
	CEBPZOS	GVINP1
	UBTD2	AHNAK2
	PLEKHA3	PATJ
	FBXO41	NFE4
	HOMEZ	HDLBP
	ARHGDIA	SRCAP
	PRG4	KCNA7
	KRIT1	FOXK1
	C17ORF107	SARS2
	PTDSS1	DMXL1
	CFAP53	PRAG1
	DSG2	GNPAT
	WDR72	PXDN
	SPRR2G	DIP2A
	EEF1B2	FEZF2
	MRI1	OLFM4
	LSR	INCENP
	ABCE1	DSCAML1
	SPP2	TAGAP
	EGFR	MAP4K4
	NDE1	PPM1D
	TNIP1	UTRN
	EXD2	ASCC3
	SIDT2	RPTN
	MTMR7	KLHL34
	TSPAN10	ANKRD18A
	PARP2	EXPH5
	LUZP1	PDE4D
	DENND2C	RSAD1
	OR6B1	ASPRV1
	STIP1	AGER
	ZNF790	SDHB
	KIAA0586	CCDC186

Only in $^{C92}EV_{UC}$	Only in $^{C92}EV/LV_{UC}$	In both $^{C92}EV_{UC}$ and $^{C92}EV/LV_{UC}$
	MAP4K3	PRAMEF20
	KIAA2026	GOLGA6C
	LIMS1	PPP1R12A
	TACC3	COL5A2
	VASH2	NFKBIE
	MSRB2	CD6
	CCL8	GCSH
	ZNF394	KRT74
	ARFGEF3	KRT84
	INPP5B	GTF3C5
	ELAC1	MPDZ
	GTPBP2	KIAA1671
	HDAC2	ACSF2
	SLC14A1	GUSB
	RPS15	C2ORF16
	GOLGA1	IQSEC1
	DHH	COX4I2
	CCDC144B	UBAP1
	LTBP1	USP37
	SLCO1B1	MRPL52
	BOD1L1	RPH3A
	IL10	STOX2
	HIP1R	IQCC
	PTCH2	ZFP28
	SFXN4	STX18
	PSG1	RUFY1
	TFB2M	CROCC2
	HAUS5	PIK3C2B
	MRPL50	LAMA1
	ROCK2	STN1
	RIPK1	UNC80
	MIF	EHMT1
	RPL31	DHX36
	PCSK1N	ARNTL
	UMPS	CBX8
	NCKAP5L	MVK
	VTI1B	REV1
	P2RX3	CUEDC1
	ERAP1	WWC3
	MED17	ALPL
	ITPKB	SIPA1L1
	FAM228A	RNASEH2B
	CD276	PASK
	BAIAP2L1	SYNRG
	TDRD3	FAM200A
	PRORSD1P	TH
	MYRFL	MOCOS
	NOS1	POLN
	UTP14A	PRODH
	DCLRE1C	TMCO5B
	SLC35F5	ARNTL2
	NXPE2	ENAH
	RPL18A	UHRF1
	SMG1	ZBED9
	SPESP1	MPC2
	BHLHE40	DGKG
	SOWAHA	FAT2
	WNK2	GALNT11

**Only in $^{C92}EV_{UC}$**	**Only in $^{C92}EV/LV_{UC}$**	**In both $^{C92}EV_{UC}$ and $^{C92}EV/LV_{UC}$**
	RPL5	BANK1
	PHF20L1	STIM1
	CCNH	EPB41L2
	FER	STAG1
	ANKRD20A12P	C2CD3
	TNFRSF8	RRP15
	CFAP46	JUP
	KLF7	PTCD1
	BIN3	CD99
	DNAJC27	TBCB
	OR10V1	S100A7
	KLHL24	CSN3
	GHSR	LYZ
	TRIM61	PGAM2
	RPL37A	HECTD1
	STYX	PRR14L
	MATR3	FRRS1L
	SOD1	FAM129A
	GABRB3	CDK7
	PLEKHA6	CHSY1
	TUBGCP6	CPT2
	VILL	COX10
	ST3GAL3	MEIKIN
	DOCK8	TSPAN6
	PARP9	REEP1
	AKAP5	DOK7
	FABP5	FAM47DP
	ARRB1	TXN
	FAM220BP	PAICS
	GCFC2	IDI1
	ZNF263	CSRP3
	SPATA21	KIAA1211
	TTLL6	AGAP2
	NSD1	MAPKAP1
	ACOT11	SLCO2A1
	DHX38	GLTPD2
	GTF2H3	CCDC112
	NCAM2	LAD1
	TNPO1	SHOC2
	ARPIN	DEFB112
	UNC119B	ERGIC3
	DDX12P	NCAPG
	IL16	RSRC2
	FAM171A2	MPEG1
	CNTNAP3B	LRRC75B
	ELP5	TUBE1
	ENKUR	TMEM205
	RAB13	EPHA8
	ZNF75A	CSN2
	CDH11	CCDC92
	CACNA1A	ARHGAP20
	MFSD4A	TSEN34
	SLC23A2	RGS1
	CCDC51	TXNDC2
	ACTRT3	CCDC126
	SYTL5	RAG2
	PSMG3-AS1	PRR14
	SMC6	CCDC136

Only in $^{C92}EV_{UC}$	Only in $^{C92}EV/LV_{UC}$	In both $^{C92}EV_{UC}$ and $^{C92}EV/LV_{UC}$
	NMNAT1	TTC19
	MCCC2	FUNDC2
	ITIH3	KIF24
	OR10C1	IGLC2
	RRP7BP	BICC1
	TSPYL1	KIAA0513
	RRAGA	DACT3
	SNUPN	CATSPERE
	DPEP1	AIFM3
	DVL2	ZNF106
	SCAF8	HAS3
	M1AP	IFIT2
	MAGED1	GAS1
	ZDHHC2	ADAMTS20
	OR2S2	PAG1
	GPSM2	CETN2
	GZMK	MEN1
	PACSIN3	SMARCAD1
	MEF2B	FAM129B
	GAPDHS	KCNH4
	ROBO1	FOXP1
	CSRP1	RTN4RL1
	RAB7B	ABTB2
	MLF1	SP100
	CYP51A1	DNALI1
	MRPL45	ZNF285
	ZNF480	MMAB
	ZFYVE21	DMKN
	RBBP8NL	RNF212B
	OSBPL7	SORCS3
	POLD3	CENPQ
	OR6V1	GSC2
	SMTN	CTDSPL2
	TMEM164	LRRC74B
	NXPH3	ALDH1A3
	PHF5A	MYL7
	VPS53	CDC42BPG
	EFR3A	ITPR1
	GRM4	ZMYM6
	ANKRD50	EPS15
	RPL36A	GSTA5
	SCG3	APOBEC3A
	CRY1	WDR90
	COL7A1	TAF4B
	ZCCHC17	LRRC17
	SYTL2	TEX22
	PPT2	PAPOLG
	ATOH1	MOCS1
	CDK16	ZFC3H1
	SNX32	TRAK2
	NOL4L	PAK1
	TEX9	TMEM26
	TIMM23B	CLPP
	KIF18A	PDK1
	ABCG2	NUP107
	EXD1	POLR3G
	NBAS	LARP7
		ACAN

Only in $^{C92}EV_{UC}$	Only in $^{C92}EV/LV_{UC}$	In both $^{C92}EV_{UC}$ and $^{C92}EV/LV_{UC}$
		TPH2
		KCNK10
		KRBA1
		THAP4
		LHCGR
		ANKRD30B
		DUPD1
		CXORF21
		CCDC124
		ACHE
		ECE1
		GLG1
		OPLAH
		GPKOW
		MADD
		INIP
		ATP1B3
		ZNF99
		SIVA1
		PNPLA6
		BCCIP
		DHX40
		SVEP1
		KAZALD1
		NBPF6
		SCN7A
		SLC45A4
		TMEM200A
		WDR73
		AVIL
		CHD1
		HABP2
		HELZ2
		DOCK3
		PCP4L1
		MAJIN
		TMEM65
		URB1
		B3GLCT
		SSBP1
		URI1
		FKBP4
		GSN
		RPS6KB1
		FAM208B
		TNRC6C
		MLF2
		TGM7
		BZW2
		TBX4
		CAPN1
		RS1
		SUFU
		ITGAX
		PRELID2
		MEIS1
		GATB
		ZBTB24

Only in $^{C92}EV_{UC}$	Only in $^{C92}EV/LV_{UC}$	In both $^{C92}EV_{UC}$ and $^{C92}EV/LV_{UC}$
		CD37
		SCRT2
		SND1
		RASD1
		FAM216B
		CCDC62
		AIFM2
		NDRG1
		CD5
		GMPPB
		AQR
		NOSTRIN
		UVSSA
		SH3TC1
		EFHC2
		PDE6B
		NEDD4L
		AHRR
		SEH1L
		FGF23
		PSMC1
		ICE1
		LINC01587
		MEX3A
		ADAMTS6
		FECH
		OR2AT4
		POM121L2
		HEATR5A
		OAS3
		CWC22
		DMWD
		SRSF7
		TBC1D8B
		ZNF840P
		TLR8
		GNG11
		UBLCP1
		GOLGA6L7
		LRRC73
		NPHS1
		FAH
		CSF3
		CTH
		NANOGNB
		CHCHD6
		BCR
		CFAP300
